# Cancer Diagnoses and Deaths in Hungary, 2011–2023: Nationwide Trends Before, During, and After the COVID-19 Pandemic

**DOI:** 10.3390/cancers18132027

**Published:** 2026-06-23

**Authors:** Zoltán Kiss, Tamás G. Szabó, Anikó Maráz, György Rokszin, Zsolt Horváth, Péter Nagy, Zsolt Abonyi-Tóth, Valéria Kovács, Orsolya Surján, Zsófia Barcza, István Kenessey, András Wéber, István Wittmann, Gergő Attila Molnár, Natali Neuhauser, Miklós Darida, István Köveskúti, Renáta Bertókné Tamás, Krisztina Bogos, Judit Moldvay, Gabriella Gálffy, Lilla Tamási, Veronika Müller, Zoárd T. Krasznai, Zsolt Pápai-Székely, Eszter Baltás, Rolland Péter Gyulai, Katalin Boér, Péter Holló, Judit Kocsis, Szabolcs Máté, Alíz Nikolényi, Zoltán Novák, Gábor Rubovszky, Magdolna Dank, Zoltán Vokó

**Affiliations:** 1 MSD Pharma Hungary Ltd., 1095 Budapest, Hungary; tamas.szabo@msd.com (T.G.S.); natali.neuhauser@msd.com (N.N.); miklos.darida@msd.com (M.D.); istvan.koveskuti@msd.com (I.K.); 2 Second Department of Medicine and Nephrology-Diabetes Centre, University of Pécs Medical School, 7624 Pécs, Hungary; wittmannistvan@gmail.com (I.W.); molnar.gergo@pte.hu (G.A.M.); 3 Department of Oncotherapy, University of Szeged, 6720 Szeged, Hungary; dr.aniko.maraz@gmail.com (A.M.); nikolenyi.aliz@gmail.com (A.N.); 4 RxTarget Ltd., 5000 Szolnok, Hungary; rokszin.gyorgy@rxtarget.hu (G.R.); abonyi-toth.zsolt@rxtarget.hu (Z.A.-T.); kovacs.valeria@rxtarget.hu (V.K.); 5 Department of Oncology, Bács-Kiskun County Teaching Hospital, 6000 Kecskemét, Hungary; zsodicsom@gmail.com; 6 Department of Molecular Immunology and Toxicology and the National Tumor Biology Laboratory, National Institute of Oncology, 1122 Budapest, Hungary; peter.nagy@oncol.hu; 7 Department of Anatomy and Histology, HUN-REN–UVMB Laboratory of Redox Biology Research Group, University of Veterinary Medicine, 1078 Budapest, Hungary; 8 Chemistry Institute, University of Debrecen, 4032 Debrecen, Hungary; 9 Department of Biostatistics, University of Veterinary Medicine, 1078 Budapest, Hungary; 10 National Center for Public Health and Pharmacy, 1097 Budapest, Hungary; surjan.orsolya@nngyk.gov.hu (O.S.); tamas.renata@nngyk.gov.hu (R.B.T.); 11 Syntesia Medical Communications Ltd., 1065 Budapest, Hungary; info@syntesia.hu; 12 Hungarian National Cancer Registry and National Tumor Biology Laboratory, National Institute of Oncology, 1122 Budapest, Hungary; kenessey.istvan@oncol.hu (I.K.); andrasvonweber@gmail.com (A.W.); 13 Department of Pathology, Forensic and Insurance Medicine, Semmelweis University, 1091 Budapest, Hungary; 14 Department of Anatomy, Cell and Developmental Biology, Eötvös Loránd University, 1117 Budapest, Hungary; 15 National Korányi Institute of Pulmonology, 1121 Budapest, Hungary; bogos@koranyi.hu; 16 First Department of Pulmonology, National Korányi Institute of Pulmonology, 1121 Budapest, Hungary; drmoldvay@hotmail.com; 17 Pulmonology Clinic, University of Szeged Albert Szent-Gyorgyi Medical School, 6720 Szeged, Hungary; 18 Translational Oncopulmonology Research Group, Institute of Molecular Life Sciences, HUN-REN Research Centre for Natural Sciences, 1117 Budapest, Hungary; 19 Pulmonology Center of the Reformed Church in Hungary, 2045 Törökbálint, Hungary; ggalffy@hotmail.com; 20 Faculty of Economics, Health Sciences and Social Studies, Károli Gáspár University of the Reformed Church in Hungary, 1088 Budapest, Hungary; 21 Department of Pulmonology, Semmelweis University,1083 Budapest, Hungary; tamasi.lilla@semmelweis.hu (L.T.); muller.veronika@semmelweis.hu (V.M.); 22 Faculty of Medicine, Department of Obstetrics and Gynecology, University of Debrecen, 4032 Debrecen, Hungary; krasznai.zoard@med.unideb.hu; 23 Fejér County Szent György University Teaching Hospital, 8000 Székesfehérvár, Hungary; zspsz64@gmail.com; 24 Department of Dermatology and Allergology, Albert Szent-Györgyi Medical School, University of Szeged, 6720 Szeged, Hungary; baltas.eszter@med.u-szeged.hu (E.B.); gyulai.rolland.peter@szte.hu (R.P.G.); 25 Department of Oncotherapy, Albert Szent-Györgyi Medical School, University of Szeged, 6720 Szeged, Hungary; 26 Department of Medical Oncology, Szent Margit Hospital, 1032 Budapest, Hungary; katalin.boer@t-online.hu; 27 Department of Dermatology, Venereology and Dermatooncology, Semmelweis University, 1085 Budapest, Hungary; hollo.peter@semmelweis.hu; 28 Department of Oncoradiology, Bács-Kiskun County Hospital, 6000 Kecskemét, Hungary; kocsisjucidr@gmail.com; 29 Department of Obstetrics and Gynecology, Semmelweis University, 1088 Budapest, Hungary; dr.mate.szabolcs@gmail.com; 30 Department of Gynecology, National Institute of Oncology, 1122 Budapest, Hungary; zoltannovak75@gmail.com; 31 Department of Thoracic and Abdominal Tumors and Clinical Pharmacology, National Institute of Oncology, 1122 Budapest, Hungary; rubovszky.gabor@oncol.hu; 32 National Tumor Biology Laboratory, 1122 Budapest, Hungary; 33 Department of Oncology, Semmelweis University, 1083 Budapest, Hungary; 34 Division of Oncology, Department of Internal Medicine and Oncology, Semmelweis University, 1083 Budapest, Hungary; foig@oncol.hu; 35 Pharmacovigilance and Patient Safety Research Group, Oncology Unit, Department of Internal Medicine and Oncology, Semmelweis University, 1082 Budapest, Hungary; 36 National Institute of Oncology, 1122 Budapest, Hungary; 37 Department of Medical Oncology, Semmelweis University, 1083 Budapest, Hungary; 38 Center for Health Technology Assessment, Semmelweis University, 1091 Budapest, Hungary; voko.zoltan@semmelweis.hu; 39 Syreon Research Institute, 1145 Budapest, Hungary

**Keywords:** COVID-19, cancer screening, pandemic impact on cancer diagnosis, cancer mortality Hungary, delayed cancer diagnosis, cancer in Hungary

## Abstract

**Background**: The coronavirus disease 2019 (COVID-19) pandemic significantly disrupted cancer screening, diagnosis, and care. This phase of the Hungarian Cancer Epidemiology (HUN-CANCER-EPI) study evaluated trends in cancer incidence and mortality in Hungary during the pre-COVID (2011–2019), COVID (2020–2021), and post-COVID (2022–2023) periods. **Methods**: Nationwide data from the Hungarian National Health Insurance Fund database were analysed. Age- and sex-adjusted incidence and mortality trends from 2011 to 2019 were modeled using Poisson regression. Changes from trends in 2020–2023 were compared to pre-COVID projections with 95% confidence intervals. **Results**: From 2011 to 2019, age-standardised cancer incidence declined by 1.9% (95% CI: 1.3% to 2.4%) annually in males and by 1.0% (95% CI: 0.6% to 1.4%) in females. During 2020–2021, incidence dropped sharply below the expected: in 2020 (−12.8% in males and −11.8% in females) and in 2021 (−11.7% and −7.9%, respectively). The largest declines affected prostate, melanoma, and kidney cancer. Rapidly progressing tumors like pancreatic and esophageal showed smaller decreases. By 2023, partial incidence rebounds were observed for prostate cancer, kidney cancer, and melanoma, likely reflecting the recovery of pandemic-delayed diagnoses. Lung and liver cancers showed no rebound. The steepest drops were in males aged 70+, with incomplete recovery. Mortality stayed near expected levels overall, with some exceptions, like melanoma, where the rebound in incidence coincided with increased mortality rates in 2023, which may reflect delayed diagnosis, although this cannot be directly confirmed. **Conclusions**: The pandemic had lasting, cancer-type-specific impacts on incidence patterns, particularly affecting screening-dependent, slow-growing tumors. Mortality remained largely stable overall during the available follow-up, highlighting the need for targeted recovery strategies and strengthened healthcare system resilience.

## Simple Summary

The COVID-19 pandemic severely disrupted cancer screening and diagnosis. Using nationwide health insurance data, this study compared cancer incidence and mortality rates before (2011–2019), during (2020–2021), and after (2022–2023) the pandemic in Hungary. The results showed steadily declining cancer incidence rates before the pandemic. During 2020–2021, incidence dropped sharply below expectations, with the largest declines seen for slow-growing tumors heavily dependent on routine screening such as prostate, kidney, and skin cancers. By 2022–2023, partial recovery was observed for some of these cancers, suggesting delayed cases were eventually detected. Lung and liver cancers showed no recovery. Overall cancer mortality remained relatively stable during the available follow-up; however, melanoma was a notable exception, where mortality increased in 2023. These findings underscore the need for targeted catch-up screening strategies and greater healthcare system resilience against future disruptions.

## 1. Introduction

The COVID-19 pandemic profoundly disrupted healthcare systems worldwide, including cancer care pathways [1]. Multiple studies have documented its impact on cancer incidence and mortality, demonstrating that diagnostic delays, reduced access to screening, and reorganized healthcare services led to a substantial decline in new cancer diagnoses, particularly during the peak years of 2020 and 2021 [2,3]. These effects were most pronounced for malignancies reliant on elective diagnostics and organized screening, such as melanoma, prostate, colorectal, and kidney cancers, while tumors with acute symptoms and rapid progression (e.g., pancreatic, esophageal, lung) showed smaller or inconsistent reductions [4].

Although international research, mainly from Western Europe and North America, has documented overall pandemic-related shifts in cancer detection and care, detailed national analyses stratified by cancer type, sex, and age remain limited. In Hungary, Elek et al. demonstrated that during 2020–2021, newly diagnosed lung, colorectal, and breast cancers decreased by 14–20% compared with expected trends, with the strongest declines observed among older age groups and some narrowing of socioeconomic disparities [5]. Another study by the same group showed that temporary reductions in organized mammography screening were associated with 15–30% fewer new breast cancer diagnoses and surgical interventions in quarters with markedly reduced screening activity compared with pre-pandemic years [6]. Notably, post-pandemic trends in cancer incidence and mortality during 2022 and 2023 are still underreported, especially in Central and Eastern Europe, where comprehensive, population-based studies spanning the entire pre-COVID (2011–2019), COVID (2020–2021), and post-COVID (2022–2023) periods are mostly lacking.

To address this gap, the HUN-CANCER EPI initiative was launched to assess cancer incidence and mortality trends in Hungary using the National Health Insurance Fund (NHIF) database which allows for the robust evaluation of both short-term disruptions and longer-term epidemiological shifts observed during the pandemic period. The current study aimed to quantify deviations in cancer incidence and mortality from pre-pandemic trends (2011–2019) during the pandemic years, examine variations across major cancer types, assess age- and sex-specific patterns, and identify rebound effects and diagnostic deficits during and after the COVID-19 pandemic (2020–2021, 2022–2023).

## 2. Materials and Methods

This nationwide, retrospective study was based on the administrative databases of the Hungarian National Health Insurance Fund (NHIF) and the Hungarian Central Statistical Office (HCSO). The NHIF database includes over 95% of the entire Hungarian population and provides detailed longitudinal records on in- and outpatient care, reimbursed medical procedures, prescribed medications, and mortality data. Diagnostic information is recorded according to the International Statistical Classification of Diseases and Related Health Problems, 10th Revision (ICD-10). The HCSO database served as the official source for annual population sizes and cause-specific mortality data by age and sex [7].

We identified patients diagnosed with malignant tumors (ICD-10 codes C00-C97, excluding C44) between 1 January 2009, and 31 December 2023. Patients were classified into cancer groups according to the ICD-10 coding system, following the grouping system defined in Ferlay’s publications [8,9]. Individuals were included in the primary incident cancer population if they had at least two occurrences of the same ICD-10 ‘C’ code (within the study period) in either inpatient or outpatient specialist care after 1 January 2011, and no cancer-related ICD-10 code during the screening period (1 January 2009, to 31 December 2010). As an exception, patients were also included if only a single C-code occurrence was recorded and any of the following conditions applied: (i) the patient died within 60 days following the first appearance of the code; (ii) the cancer-related ICD-10 code was recorded as the cause of death in the NHIF mortality data; (iii) at least one oncology-specific intervention was associated with the cancer ICD-code (e.g., systemic anticancer therapy, radiotherapy, oncologic surgery, or a valid morphology code). Where more than one cancer-related ICD-10 code was recorded, the tumor type with the highest number of occurrences (over the whole study period) was selected as the index primary cancer, unless a different cancer type met the stricter inclusion criteria outlined above. The date of diagnosis was defined as the earliest date on which the relevant C-code appeared. Patients with second or third primary cancers were also identified using a standardized algorithm. A second primary cancer was defined as a distinct Ferlay-classified tumor diagnosed at least 12 months after the index primary cancer, involving at least two occurrences of a different ICD-code and supported by at least one oncology-specific intervention. Similarly, a third primary cancer was defined as a further, new ICD-code distinct from both primary and secondary cancers, also observed at least one year later and linked to at least one oncology-specific procedure. Only cases that met all criteria for subsequent primary cancers were included. ICD-10 codes of C44 (non-melanoma skin cancer) were excluded in accordance with international cancer epidemiology standards. For international comparisons, we also calculated total cancer incidence by summing cases from all eligible ICD-10 categories (excluding C44). For cervical cancer specifically, two cancer-related ICD-10 codes were required within 30 to 180 days, based on the results of sensitivity analyses. As ICD coding practices for cervical cancer changed during the study period, precancerous lesions were no longer routinely coded as invasive cervical cancer.

Sensitivity analyses were performed to assess the robustness of our case definitions and to explore how alternative assumptions might influence the observed incidence trends. Because the NHIF claims database does not contain individual-level clinical validation, we relied on administrative proxies to test the plausibility of our identification algorithm. First, we compared the primary case definition with alternative definitions that were either stricter or more permissive with respect to the number of required ICD-10 code occurrences and the time interval allowed between repeated claims. This allowed us to examine whether the observed trends were driven by the exact coding rules used for case ascertainment. Second, we assessed the proportion of identified cases that could be corroborated by oncology-specific evidence, including surgery, radiotherapy, systemic anticancer treatment, or morphology codes. In addition, we examined consistency with cause-specific mortality data from the NHIF to further support the plausibility of the identified cases at the population level. Across these alternative specifications, the main findings remained materially unchanged, suggesting that the observed incidence patterns were not highly dependent on a single operational definition of a cancer case.

Annual age- and sex-specific population sizes and cause-specific mortality data were obtained from the CSO. The number of newly diagnosed cancer cases each year was expressed as absolute numbers (n), and annual incidence and cause-specific mortality rates were calculated as standardized rates per 100,000 person-years (PYs). Age-standardized rates were computed using the European Standard Population (ESP) 2013 weights [10]. Wherever the crude number of individuals in a category was below ten, the value was masked in accordance with NHIF data protection policies; in such cases zero cases were imputed for calculations concerning the data points. For cause-specific mortality, the data masking threshold was lower (3 cases), and it was possible to differentiate from layers with 0 cases. A virtual case number of 1.5 was imputed for these data point to enable downstream statistical analyses.

Annual incidence and mortality trends were evaluated using Poisson regression models. Calendar year was included as a continuous linear predictor to estimate the underlying pre-pandemic trend, while sex and age group (10-year bandwidth) were included as covariates. Deviations during the pandemic and post-pandemic years were modeled using year-specific indicator variables. The log-transformed population size was included as an offset. Poisson regression was chosen because the outcomes were annual counts of incident cancer cases and deaths, making this approach appropriate for modeling rate data with population size included as an offset. The pre-pandemic period (2011–2019) was used to estimate the underlying secular trend with a simple linear time term as an operational and interpretable approximation of long-term change. The assumption of approximate linearity in the pre-pandemic trend was evaluated descriptively and supported by the sensitivity analysis using alternative age-group specifications. Because the number of cases below age 30 was low for most studied diagnoses, and because data masking introduced substantial noise in the 0–29 age group, these observations were excluded from the primary statistical model. This decision was also consistent with the study focus on adult-onset cancers, for which the youngest age groups were not central to the research question. To examine whether the choice of 10-year age bands and the masking of small cell counts had any meaningful influence on the estimated trends, we fitted an alternative model using single-year age bins and unmasked case numbers. The results of this sensitivity analysis are shown in Supplementary Table S1. The overall findings were materially unchanged, indicating that the primary results were robust to this alternative model specification.

We defined 2011–2019 as the pre-pandemic period, 2020–2021 as the COVID period and 2022–2023 as the post-COVID period, acknowledging that healthcare recovery was gradual. A single statistical model was built to simultaneously estimate a long-term trend (data from 2011–2019) and the deviation of rates in individual years from the trend starting with 2020, adding those years as extra categorical variables. Results are shown as the estimated difference between values observed during the COVID period (2020–2021) or post-COVID period (2022–2023) and expectations based on the average annual percent change (AAPC) in the 2011–2019 period. The differences were expressed as relative deviations in percent with 95% confidence intervals (95% CIs). Post-pandemic rebound was defined as incidence that was not significantly below the incidence expected based on the 2011–2019 trend. To address potential bias from repeated measurements across years, CIs were calculated using the robust sandwich estimator from the sandwich package in R. A *p*-value of less than 0.05 was considered statistically significant. All analyses were performed in R version 4.2.1 (https://www.r-project.org) (Accessed date: 30 November 2025).

The study was approved by the Hungarian National Ethical Committee (IV/298-2/2022/EKU).

## 3. Results

### 3.1. Trends in Overall Cancer Incidence and Mortality by Age and Sex in Hungary, 2011–2023

**Figure 1:**
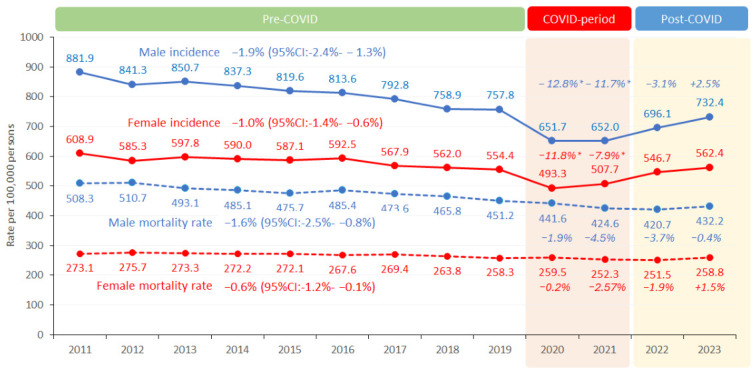
Age-standardized incidence and mortality rates of all cancer types (excluding C44) in Hungary between 2011 and 2023 (ESP 2013), with percentage changes shown relative to the expected trend based on the pre-COVID period (2011–2019). The figure highlights changes in incidence during the COVID period (2020–2021) and in the post-COVID years (2022–2023), alongside diverging mortality trends by sex (* relates to significant changes). ESP, European Standard Population.

Between 2011 and 2019, age-standardized cancer incidence and mortality rates (Supplementary Tables S2–S5) showed a consistent decline in Hungary for both sexes. In males, cancer incidence decreased by an average of 1.9% per year (95% CI: −2.4% to −1.3%), while females experienced a slower decline of 1.0% annually (95% CI: −1.4% to −0.6%) (Figure 1). Mortality rates followed a similar downward trend, with annual decreases of 1.7% (95% CI: −2.5% to −0.9%) for males and 0.7% (95% CI: −1.2% to −0.1%) for females. In 2020, observed cancer incidence dropped sharply below expected levels in both sexes: by 12.8% (95% CI: −16.6% to −8.9%) in males and by 11.8% (95% CI: −15.9% to −7.7%) in females (Figure 1). This reduction persisted in 2021, with incidence rates remaining 11.7% (95% CI: −17.7% to −16.6%) lower than expected in males and 7.9% (95% CI: −11.5% to −4.3%) lower in females. By 2022–2023, incidence trends diverged by sex. A rebound in incidence was observed in both sexes, although its magnitude and statistical significance varied by cancer type and sex. Specifically, females demonstrated a statistically significant increase above expected levels in 2023, with incidence exceeding predictions by 4.8% (95% CI: 0.5% to 9.2%).

**Figure 2:**
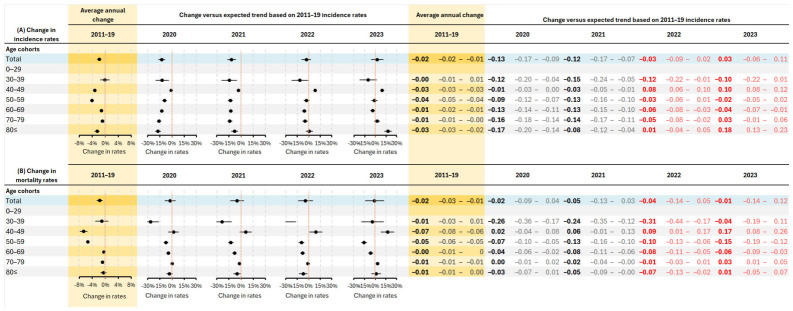
Annual changes in age-specific overall cancer incidence (**A**) and mortality (**B**) rates among males in Hungary during the pre-COVID period (2011–2019), and percentage deviations from the expected trends in the COVID (2020–2021) and post-COVID (2022–2023) periods. Expected values were projected based on linear trends from the pre-pandemic years.

Mortality remained close to predicted values from 2020 to 2023 in both sexes (Figure 1 and Figure 2), consistent with the long-term declining trend observed across the study period. However, delayed consequences of pandemic-related diagnostic disruptions may only become apparent in subsequent years.

**Figure 3:**
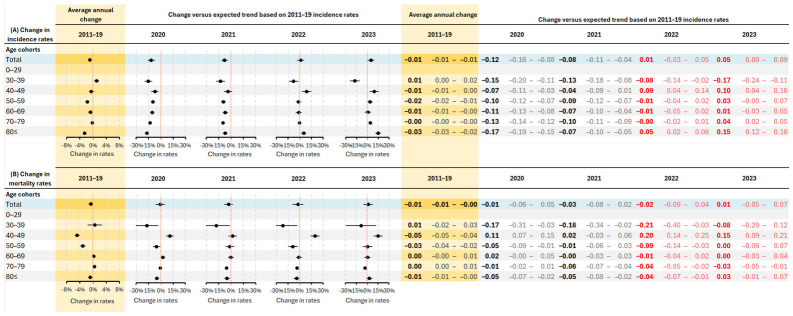
Annual changes in age-specific overall cancer incidence and mortality rates among females in Hungary during the pre-COVID period (2011–2019), and percentage deviations from the expected trends in the COVID (2020–2021) and post-COVID (2022–2023) periods. Expected values were projected based on linear trends from the pre-pandemic years.

Age-specific trends in the pre-pandemic period revealed the largest incidence and mortality decreases in middle-aged groups, particularly in males aged 40–49 years (incidence change: −3.2% [95% CI: −3.4% to −3.0%]; mortality change: −6.6% [95% CI: −7.7% to −5.5%]) and 50–59 years (incidence change: −4.1% [95% CI: −4.6% to −3.6%]; mortality change: −5.3% [95% CI: −5.7% to −4.9%]) (Figure 2). Women showed comparable declines in these age groups (Figure 3). Notably, females aged 30–39 years experienced a slight increase in incidence (1.1% [95% CI: 0.4% to 1.9%]) without a corresponding rise in mortality.

During the COVID period, males exhibited an age-dependent decline in incidence, which was especially pronounced in the oldest cohort (80+ years: −17.3% [95% CI: −20.4% to −14.1%] in 2020), with some rebound observed by 2023, notably in the 40–49 and 80+ age groups (3.4% [95% CI: 7.5% to 11.9%] and 17.7% [95% CI: 12.7% to 22.7%], respectively) (Figure 2). Male mortality rates were generally lower than expected, except for an increase in the 70–79 age group in 2023 (3.3% [95% CI: 1.1% to 5.5%]). Females experienced a similar initial decline in incidence, with substantial rebound in 2023 concentrated in the 30–39 and 80+ age groups (9.5% [95% CI: 3.5% to 15.5%] and 14.9% [95% CI: 11.5% to 18.3%], respectively). Female mortality remained stable, except for a significant increase in the 40–49 age group (14.9% [95% CI: 9.7% to 20.7%] in 2023), indicating a deceleration of previous improvements.

In summary, the COVID-19 pandemic was associated with a significant transient decline in cancer incidence without corresponding changes in mortality. This pattern is compatible with widespread diagnostic delays during the pandemic, although a direct assessment of delayed diagnoses was beyond the scope of this study. Post-pandemic rebounds in incidence rates, particularly among females, highlight the need for ongoing monitoring and healthcare system adaptation.

### 3.2. Trends in Cancer Incidence and Mortality by Cancer Type (2011–2023)

#### 3.2.1. Males

**Figure 4:**
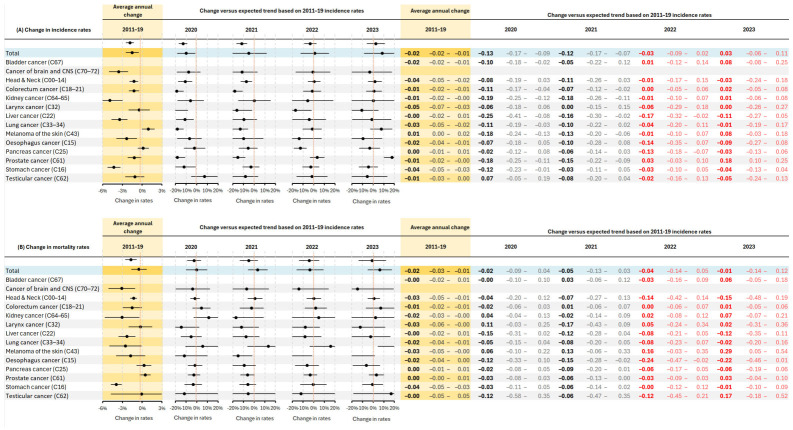
Annual changes in cancer incidence (**A**) and mortality (**B**) rates by main cancer types among males in Hungary during the pre-COVID period (2011–2019), and percentage deviations from the expected trends in the COVID (2020–2021) and post-COVID (2022–2023) periods. Expected values were projected based on linear trends from the pre-pandemic years.

Figure 4 shows a detailed breakdown of male cancer incidence trends by cancer site during the entire study period. The incidence of lung cancer showed a steady pre-pandemic decline of 3.4% annually (95% CI: −4.6% to −2.3%). This decline accelerated during the pandemic years with a significant incidence reduction of 11.1% (95% CI: −19.2– to 3.0%) in 2020 and consistent, although not significant reductions in 2021–2023. Laryngeal and head and neck cancers also exhibited significant annual decreases pre-COVID (change: −4.9% [95% CI: −6.8% to −3.0%] and −3.6% [95% CI: −5.0% to −2.2%], respectively), with smaller and not statistically significant pandemic-related dips (estimated decreases ranging from 7.4% to 9.1%). Esophageal cancer showed a milder pre-pandemic decline (change: −2.3%/year [95% CI: −3.9% to −0.8%]), and was only marginally affected by the pandemic, maintaining relatively stable incidence rates. In contrast, prostate cancer demonstrated a decreasing incidence trend before the pandemic (change: −1.2%/year [95% CI: −2.2% to −0.2%]), followed by substantial incidence reductions of 18.1% (95% CI: −25.2% to −11.1%; 2020) and 15.3% (95% CI: −21.9% to −8.6%; 2021), and a rebound of 17.7% (95% CI: 10.2% to 25.3%) in 2023. Melanoma incidence in males increased modestly pre-COVID (0.9% [95% CI: 0.0% to 1.8%] per year), dropped sharply during the pandemic (change: −18.1% [95% CI: −23.7% to −12.6%] in 2020, −13.0% [95% CI: −20.1% to −6.0%] in 2021), and then showed not yet significant increases, showing a rebound above expected levels. Kidney cancer incidence followed a similar pattern, with sharp pandemic declines and minimal recovery, suggesting persistent diagnostic delays. Rapidly progressing tumors such as pancreatic cancer and liver cancer did not show significant incidence rebounds post-pandemic. Pancreatic cancer incidence remained largely unchanged during the pandemic, with a slight downward trend continuing in 2022 and 2023. Liver cancer incidence also declined slightly during the pandemic and remained stable thereafter.

Cancer mortality trends in males largely mirrored incidence changes. Pre-COVID mortality for lung cancer decreased by approximately 2.3% (95% CI: −3.6% to −1.1%) annually. In 2023, melanoma mortality increased significantly by 9.3%, consistent with delayed diagnosis leading to more advanced disease stages at presentation. Prostate cancer mortality showed a non-significant upward trend in 2023 (3.1%).

#### 3.2.2. Females

**Figure 5:**
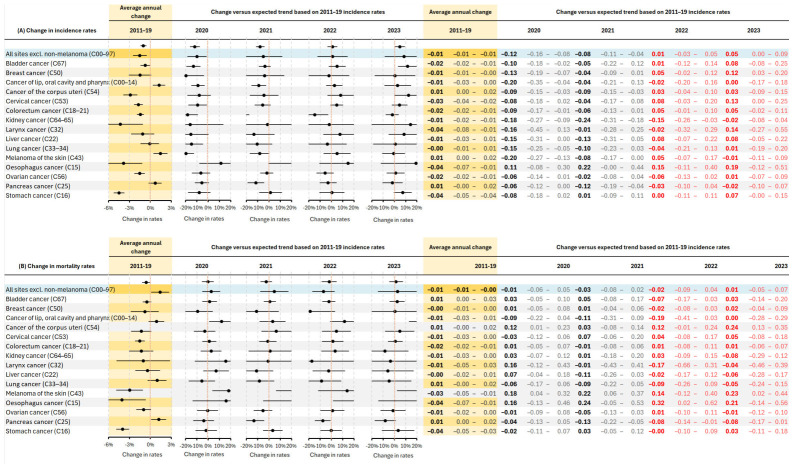
Annual changes in cancer incidence (**A**) and mortality (**B**) rates by main cancer types among females in Hungary during the pre-COVID period (2011–2019), and percentage deviations from the expected trends in the COVID (2020–2021) and post-COVID (2022–2023) periods. Expected values were projected based on linear trends from the pre-pandemic years.

The analysis of female cancer incidence stratified by cancer type (Figure 5) revealed a modest pre-pandemic decline in breast cancer incidence (change: −0.7%/year [95% CI: −1.4% to −0.1%]), followed by a marked incidence drop during the pandemic (change: −13.0% [95% CI: −19.1% to −7.0%] in 2020), and a sharp rebound of 11.7% (95% CI: 3.4% to 20.0%) in 2023, reaching statistical significance. Cervical cancer incidence similarly declined pre-pandemic (change: −2.9%/year [95% CI: −3.8% to −1.9%]), with a non-significant pandemic-related decrease, and a notable rebound of 12.6% (95% CI: 0.1% to 25.1%) in 2023. Ovarian cancer incidence showed steady declines both before and during the pandemic, with no clear rebound. Melanoma incidence among females increased pre-pandemic (1.5% [95% CI: 0.5% to 2.4%] annually) but was disrupted by a sharp decline in 2020 (change: −19.7% [95% CI: −26.7% to −12.8%]) and a smaller decline in 2021 (change: −8.4% [95% CI: −16.9% to 0.1%]), before modest and not statistically significant rebounds in 2022 and 2023. Kidney cancer incidence in females declined sharply during the pandemic (change: −18.1% [95% CI: −26.8% to −9.4%] in 2020 and −24.4% [95% CI: −31.2% to −17.6%] in 2021), without post-pandemic recovery.

Female cancer mortality rates demonstrated stable or slightly declining trends pre-pandemic, particularly for breast and cervical cancers, consistent with improved detection and treatment. During the pandemic years, mortality trends remained largely stable, with no significant excess mortality observed for most cancer types. However, despite the absence of significant changes in the incidence or rebound for uterine cancer, its mortality rate was substantially higher than expected during the entire pandemic and post-pandemic period. The observed mortality increase was 11.8% (95% CI: 1.0% to 22.5%) in 2020, with fluctuations continuing through 2023 and an overall excess mortality of approximately 24.2% (95% CI: 13.0% to 35.4%) over the pre-pandemic trend. Unexpectedly and in contrast to men, melanoma mortality in women already increased significantly by 18.2% (95% CI: 3.9% to 35.2%) in 2020, with elevated mortality persisting through 2023 (overall increase: 23.0% [95% CI: 2.5% to 43.5%]). In 2023, a slight increase in breast cancer mortality (2.5%) was also noted, although this did not reach statistical significance. Cervical cancer mortality rates remained stable throughout the study period.

### 3.3. Age-Dependent Changes in the Pre-COVID, COVID and Post-COVID Periods for the Most Common Cancer Types

#### 3.3.1. Lung Cancer

**Figure 6:**
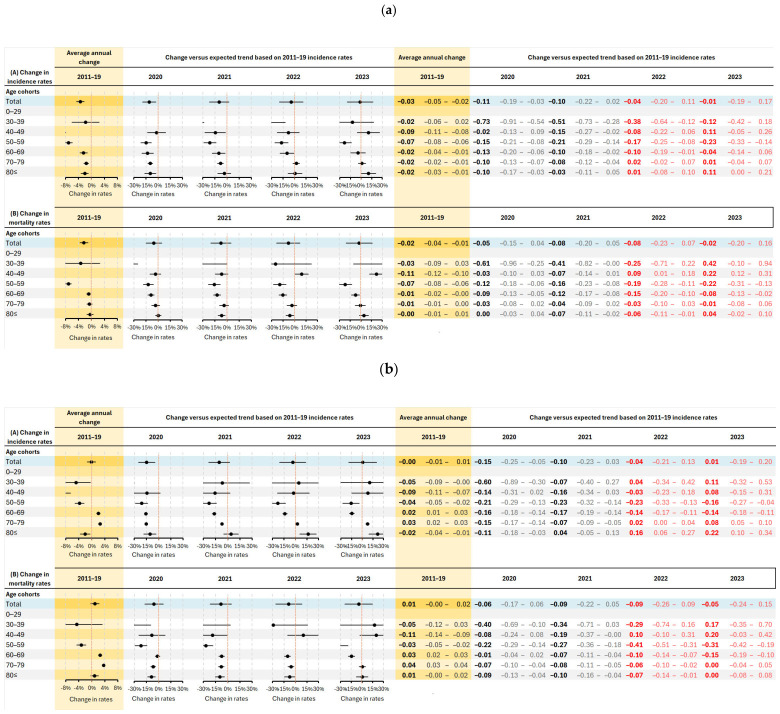
Annual changes in age-specific lung cancer (C33-34) incidence and mortality rates among males (**a**) and females (**b**) in Hungary during the pre-COVID period (2011–2019), and percentage deviations from the expected trends in the COVID (2020–2021) and post-COVID (2022–2023) periods. Expected values were projected based on linear trends from the pre-pandemic years.

The most pronounced declines in lung cancer incidence before the COVID-19 pandemic (2011–2019) were observed in the age groups of 40–49 and 50–59 years in males, with annual decreases of 9.5% (95% CI: −11.0% to −8.0%) and 7.0% (95% CI: −8.1% to −5.9%), respectively (Figure 6a). Among females, the 40–49 age group also showed a significant annual decline of 8.7% (95% CI: −10.8% to −6.5%), while incidence declined by 3.7% (95% CI: −5.1% to −2.3%) in the 50–59 age group (Figure 6b). During the first two pandemic years (2020–2021), lung cancer incidence dropped sharply, most notably in the 50–59 age group among males, with decreases exceeding 11% compared to expected values based on pre-pandemic trends. Similar patterns were seen in females, where the 50–59 cohort experienced incidence reductions of 21.0% (95% CI: −28.6% to −13.4%) in 2020 and 23.3% (95% CI: −31.9% to −14.2%) in 2021. Importantly, in the post-COVID period (2022–2023), lung cancer incidence did not return to pre-pandemic expected levels in the 50–59 age cohort. Incidence rates in these groups remained persistently lower than anticipated, with deficits of up to 4.3% (95% CI: −19.8% to 11.3%) observed in 2022 and 1.3% (95% CI: −19.5% to 17.0%) in 2023 among males. This sustained reduction was particularly evident even in mortality trends in the 40–59 age cohorts, while older cohorts showed smaller declines or slight rebounds.

#### 3.3.2. Colorectal Cancer

**Figure 7:**
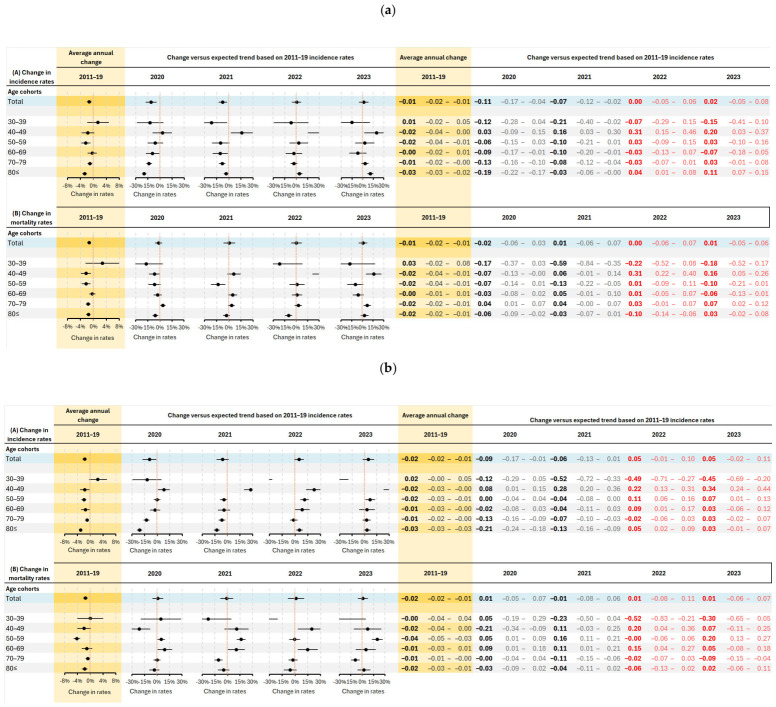
Annual changes in age-specific colorectal cancer (C18–21) incidence and mortality rates among males (**a**) and females (**b**) in Hungary during the pre-COVID period (2011–2019), and percentage deviations from the expected trends in the COVID (2020–2021) and post-COVID (2022–2023) periods. Expected values were projected based on linear trends from the pre-pandemic years.

Between 2011 and 2019, the incidence of colorectal cancer in Hungary declined steadily in both sexes (males: −1.3%/year [95% CI: −1.9% to −0.6%]; females: −1.7%/year [95% CI: −2.3% to −1.1%]) (Figure 7a,b and Figure S2). The most pronounced declines occurred in those aged 80+ years (males: −2.7%/year [95% CI: −3.3% to −2.1%]; females: −3.0%/year [95% CI: −3.4% to −2.7%]). Moderate annual decreases were seen in the 50–59 years (−2.3% [95% CI: −3.7% to −1.0%] in males; −2.0% [95% CI: −2.7% to 1.2%] in females) and 60–69 years (−1.5% [95% CI: −2.7% to −0.3%] in females) age cohorts. A non-significant increase was observed only in the 30–39 age group in both sexes (1.4% [95% CI:−1.8% to 4.7%] in males; 2.4% [95% CI: −0.4% to 5.1%] in females).

During the COVID-19 pandemic (2020–2021), colorectal cancer incidence dropped significantly compared to expected trends (males: −10.9% [95% CI: −17.4% to −4.4%] in 2020 and −7.4% [95% CI: −12.5% to −2.3%] in 2021; females: −9.2% [95% CI: −17.2% to −1.1%] and −5.7% [95% CI: −12.6% to 1.3%], respectively). Although modest increases were observed in 2022 and 2023, values remained below pre-pandemic levels (Supplementary Figure S2). In 2020, incidence dropped sharply in older age groups: 13.4% (95% CI: −16.5% to −10.3%) and 19.5% (95% CI: −21.7% to −17.2%) lower vs. expected in males aged 70–79 and 80+ years, respectively, and 12.7% (95% CI: −16.0% to −9.5%) and 21.0% (95% CI: −23.8% to −18.1%) lower than expected in females, respectively. While some rebound occurred by 2023, mainly in the 70–79 and 80+ years cohorts, recovery was partial and inconsistent. Age-specific mortality trends showed steady long-term declines in middle-aged and older groups, with the steepest reductions observed among males aged 50–59 years (−2.3%/year [95% CI: −3.5% to −1.0%]) and 80+ years (−1.5%/year [95% CI: −2.1% to −0.9%]), and in females aged 80+ years (−1.7% [95%: −2.5% to −0.9%]) and 60–69 years (−1.0% [95% CI: −2.6% to 0.5%]). In 2023, male colorectal cancer mortality increased significantly in non-screened age groups: by 15.8% (95% CI: 5.4% to 26.2%) in the 40–49 and by 6.8% (95% CI: 1.9% to 11.7%) in the 70–79 cohort. In contrast, no excess mortality was seen in the screened 50–69 age groups, where mortality was even lower (albeit, not in a statistically significant manner) than expected: 10.0% (95% CI: −20.8% to 0.8%) lower in the age group of 50–59 years and 5.8% (95% CI: −13.0% to 1.3%) lower in those aged 60–69 years.

#### 3.3.3. Breast Cancer

**Figure 8:**
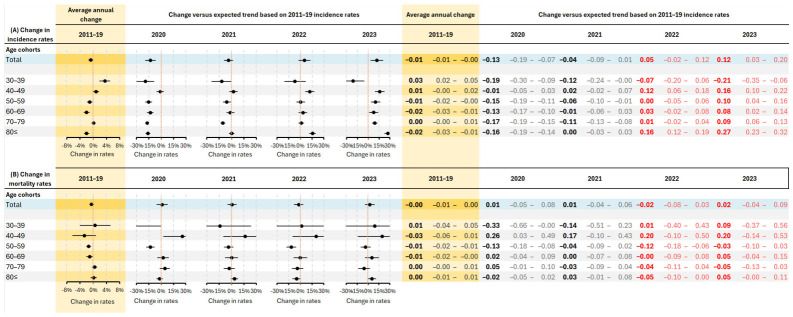
Annual changes in age-specific breast cancer (C50) incidence and mortality rates among females in Hungary during the pre-COVID period (2011–2019), and percentage deviations from the expected trends in the COVID (2020–2021) and post-COVID (2022–2023) periods. Expected values were projected based on linear trends from the pre-pandemic years.

The age-standardized incidence of female breast cancer in Hungary declined modestly between 2011 and 2019 (AAPC: −0.7% [95% CI: −1.4% to −0.1%]), dropped sharply in 2020 (−13.0% [95% CI: −19.% to −7.0%]), and showed a strong rebound in 2022 and 2023, with increases of 5.2% (95% CI: −2.0% to 12.4%) and 11.7% (95% CI: 3.4% to 20.0%), respectively, reaching the highest rates of 148.0 per 100,000 PYs during the study period (Supplementary Figure S3). In contrast, age-standardized mortality declined slightly pre-pandemic (AAPC: −0.5% [95% CI: −1.1% to 0.1%]) and showed no significant deviation from trends during 2020–2023. Prior to the COVID-19 pandemic, breast cancer incidence increased significantly by 3.5% (95% CI: 2.0% to 5.0%) per year among women aged 30–39 years (Figure 8) (from 43.1 to 51.3/100,000 PYs; Supplementary Figure S4) and by 0.9% (95% CI: 0.0% to 1.8%) per year in women aged 40–49 years. In contrast, incidence declined by 1.1% (95% CI: −1.9% to −0.4%) annually in the 50–59 age group, by 2.0% (95% CI: −2.8% to −1.2%) in the 60–69 age group and by 2.0% (95% CI: −2.7% to −1.1%) in the ≥80 age group and remained stable in women aged 70–79 years (0.1%/year [95% CI: −0.4% to 0.7%]).

The COVID-19 pandemic led to major shifts in breast cancer incidence across all age groups, with varying recovery patterns. In women aged 30–39 years, incidence dropped sharply by 19.2% (95% CI: −29.6% to −8.8%) in 2020 and remained 20.7% (95% CI: −35.5% to −5.9%) below expectations by 2023. In contrast, the 40–49 age group showed only a minor decline in 2020 (−1.2% [95% CI: −5.3% to 3.1%]), followed by a strong rebound peaking in 2023 at 177.7 per 100,000 PYs—the highest during the study period (Supplementary Figure S4). Other age groups (50–79 years) experienced steep declines in 2020 (−13.3% [95% CI: −17.0% to −9.6%] to −17.2% [95% CI: −19.2% to −15.2%]), with partial rebounds by 2023, while the 80+ cohort saw a full recovery and 27.2% (95% CI: 22.7% to 31.6%) excess incidence by 2023. Despite these fluctuations, breast cancer mortality remained stable across all age groups, with no significant pandemic-related deviations.

#### 3.3.4. Melanoma

**Figure 9:**
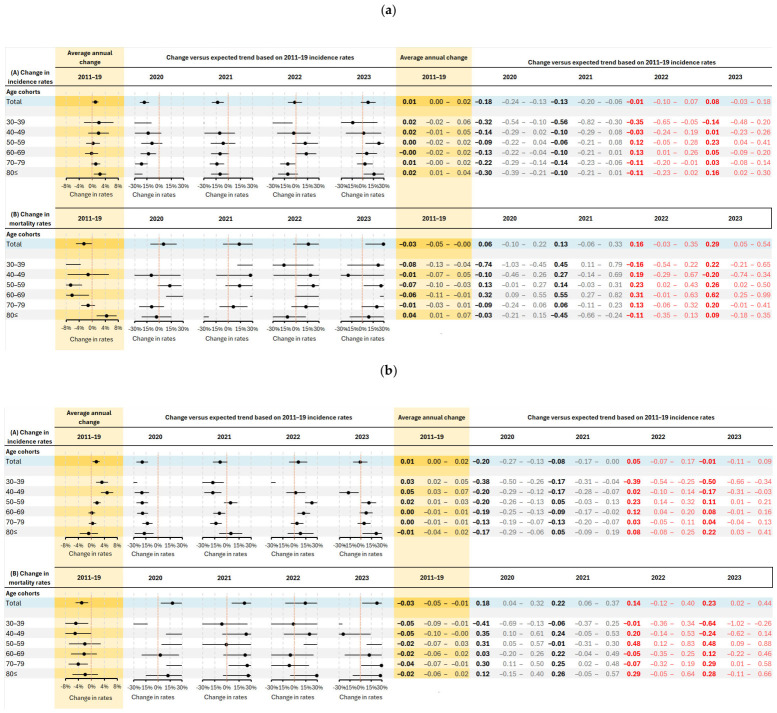
Annual changes in age-specific melanoma incidence and mortality rates among males (**a**) and females (**b**) in Hungary during the pre-COVID period (2011–2019), and percentage deviations from the expected trends in the COVID (2020–2021) and post-COVID (2022–2023) periods. Expected values were projected based on linear trends from the pre-pandemic years.

Between 2011 and 2019, melanoma incidence plateaued following previous increases, while mortality declined steadily (Supplementary Figure S5). However, the COVID-19 pandemic disrupted these trends: incidence dropped sharply in 2020–2021 but rebounded by 2023, whereas mortality, especially among women, increased significantly and remained higher than before (Figure 9a,b). During the COVID-19 period (2020–2021), melanoma incidence declined across all male age groups, with the largest decrease observed in the oldest cohort (−30.1% [95% CI: −39.5% to −20.85%] in men aged 80+ years), followed by a significant rebound in 2022–2023, particularly among men aged 50–69 years, where incidence showed the greatest excess over expectations. In women, incidence showed a declining trend pre-COVID but dropped by about 20% in nearly all age groups in 2020, and then increased significantly above expected levels in the 50–69 age group during 2022–2023. Mortality in this middle-aged female cohort also increased significantly above expected values during the COVID and post-COVID periods.

#### 3.3.5. Prostate Cancer

**Figure 10:**
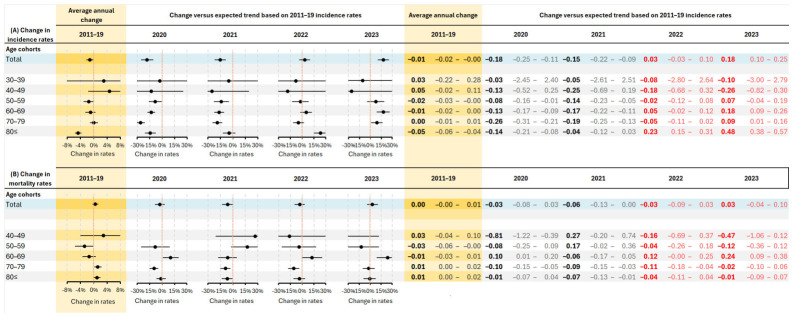
Annual changes in age-specific prostate cancer incidence and mortality rates among males in Hungary during the pre-COVID period (2011–2019), and percentage deviations from the expected trends in the COVID (2020–2021) and post-COVID (2022–2023) periods. Expected values were projected based on linear trends from the pre-pandemic years.

During the pre-COVID period (2011–2019), prostate cancer incidence showed a significant annual decrease of 1.2% (95% CI: −2.2% to −0.2%), with the most pronounced decline observed in the ≥80 age group (−4.7% [95% CI: −5.6% to −3.7%]), while mortality remained stable across all age cohorts (Figure 10 and Figure S6). In 2020 and 2021, incidence dropped significantly in all age groups: by 26.0% (95% CI: −30.6% to −21.4%) and 14.2% (95% CI: −20.6% to −7.8%) in the 70–79 and ≥80 group, respectively, with moderate declines in younger age cohorts. Mortality during the COVID years showed non-significant changes in most age groups, except for a notable 80.6% (95% CI: −122.1% to −39.2%) decrease in the 40–49 group in 2020. In 2023, a strong rebound in incidence was observed: 17.7% (95% CI: 9.2% to 26.1%) in the 60–69 group, 8.8% (95% CI: 1.1% to 16.4%) in the 70–79 group, and 47.6% (95% CI: 38.5% to 56.6%) in the ≥80 group. Mortality remained largely unchanged except in the 60–69 group, where a 23.7% (95% CI: 9.1% to 38.3%) increase was detected in 2023.

#### 3.3.6. Further Main Cancer Types Including Bladder and Pancreatic Cancer

Bladder cancer incidence in Hungary showed a significant declining trend among men between 2011 and 2019, particularly in the 50–69 age group, while female rates remained largely stable. The COVID-19 pandemic led to a sharp but temporary drop in incidence in both sexes, most prominently in younger males, suggesting pandemic-related diagnostic delays. Mortality remained stable throughout, indicating that short-term disruptions did not translate into worse outcomes. Pancreatic cancer incidence and mortality showed no meaningful long-term trend or COVID-related disruption between 2011 and 2023, with consistently high lethality and closely aligned incidence and mortality rates in both sexes. Detailed age-specific analyses and visualizations of main cancer types are presented in the Supplementary Document and Figures.

## 4. Discussion

We observed a persistent decline in Hungarian cancer incidence through both 2020 and 2021, with limited recovery in 2022, and a rebound in 2023. Besides the striking trend changes, the decline in cancer incidence observed in Hungary in 2020 appears particularly pronounced, as compared to international trends. According to recent population-based studies, the cumulative decrease in newly diagnosed malignant cases during 2020 varied across Europe, with declines reported in Sweden and Finland, while no significant reduction was observed in Denmark, Norway, or the Faroe Islands [11]. Iceland even reported a slight excess in incident cases (4.2%). In the United States, SEER-22 data showed a significant decline in overall cancer incidence in 2020, followed by a smaller decrease in 2021 [3]. Similar reductions were reported in Germany and England [12]. A cohort study from the Greater Poland region also found a substantial decrease in new cancer diagnoses during the first pandemic year [13]. Indirect evidence from other neighboring Central European countries also suggests a decline in cancer diagnoses during the pandemic period; however, no comprehensive, systematic studies are available [14,15]. Overall, these findings indicate that the pandemic affected cancer diagnosis patterns across multiple countries, although the magnitude and duration of the decline varied considerably.

While the observed decline in overall cancer incidence in Hungary during 2020 appears pronounced, drawing direct international comparisons remains challenging due to substantial heterogeneity in analytical approaches. Studies vary in the calendar periods analyzed (e.g., March–December 2020 versus the full year), the reference years used for comparison (2019 versus the 2017–2019 averages), and whether they report raw counts or age-standardized rates. Nevertheless, available data suggest that although the magnitude of the decline observed in Hungary is among the higher reductions reported across Europe (12.8% and 11.8% in 2020 for males and females), they are not exceptionally high. For example, Germany and Sweden reported comparable decreases (7.1% and 6.2%, respectively), while Poland experienced even greater drops (20% in men and 17% in women).

The Hungarian trends can also be considered in the broader context of global patterns. According to a meta-analysis by Angelini and Teglia et al., the average decline in new cancer diagnoses across Europe in early 2020 was approximately 25.4%, indicating that the Hungarian figures fall within the range of observed international variation [16]. However, unlike in some Western countries such as the United States and England where incidence rebounded or normalized by 2021, Hungary continued to show a deficit in new cancer diagnoses well into 2022, and incidence rates remained consistently below pre-pandemic levels through 2022. A rebound was observed in 2023, mainly in cancer types with high survival rates and strong dependence on screening (e.g., melanoma, breast cancer, and prostate cancer).

This pattern is also consistent with broader evidence syntheses on screening disruption and diagnostic delays. Angelin and Teglia et al. reported the smallest reduction for breast cancer and larger declines for gastrointestinal, skin, and genitourinary cancers, consistent with greater disruption of screening-dependent, high-survival tumors [16]. Similar patterns were observed in Belgium and England, where marked declines in melanoma, breast, and prostate cancer diagnoses were reported, and some cases remained undiagnosed after the first pandemic waves [17,18]. In contrast, more aggressive or symptomatic cancers such as pancreatic, esophageal, lung, and laryngeal cancers showed smaller relative declines [9,19]. Several countries have also reported incomplete rebounds in incidence for certain cancer types, raising concern that some cases remained undiagnosed [4,20]. It is also worth mentioning that some published studies raise the possibility of oncogenic potential of the SARS-CoV-2 virus itself [21,22,23].

Differences in melanoma growth kinetics by histological subtype contribute to the observed sex-specific and delayed mortality patterns. Superficial spreading melanoma (SSM), which accounts for approximately two-thirds of melanoma cases and is more frequent in women, typically shows slower growth rates, making short-term diagnostic delays less critical for survival. In contrast, nodular melanoma (NM), which contributes disproportionately to melanoma-related deaths, is characterized by rapid vertical growth, so diagnostic delays may have more serious consequences [24,25].

These findings highlight the complex interaction between cancer biology, healthcare systems, and patient behavior during and after the pandemic, and underscore the need for standardized, pan-European analyses using uniform frameworks to enable reliable cross-country comparisons and inform future disruption responses. While the general pattern of reduced incidence and partial recovery has been reported internationally, our study adds nationwide Hungarian evidence with long pre-pandemic follow-up and detailed tumor- and sex-specific analyses.

### 4.1. Age-Dependent Impact of the COVID-19 Pandemic on Cancer Incidence and Mortality

Our study reveals a clear age-dependent impact of the COVID-19 pandemic on cancer diagnosis and mortality in Hungary, with older adults, especially men aged 80 years and older, experiencing the largest declines in cancer incidence during 2020, mirroring international findings. In a UK study by Maringe et al. (2020), the reduction in cancer diagnoses during the early pandemic was more profound among older adults, reflecting both higher vulnerability to COVID-19 infection and greater disruption of routine healthcare access in this population [26]. Similarly, Dutch cancer registry data demonstrated that cancer incidence dropped most sharply among patients aged 70 and older, with some age groups experiencing up to a 25% decrease in diagnoses during 2020 compared to pre-pandemic levels [27]. The reductions may have reflected deferred screening, reduced healthcare access, and patient reluctance to seek care due to infection fears, particularly among the elderly [28,29,30]. By 2023, a notable rebound in cancer diagnoses was found in our study, primarily in age groups and cancer types associated with screening (e.g., prostate, melanoma, breast cancer), consistent with patterns observed internationally. A Swedish cohort study reported sharp declines in melanoma and prostate cancer diagnoses in 2020, most likely reflecting delayed detection due to interrupted screening and routine care. Similarly, a systematic review showed substantial diagnostic catch-up for breast cancer after pandemic-related service suspensions [31,32,33].

In our study, mortality remained stable or slightly decreased in most groups during the pandemic, although increases were seen in males aged 70–79 and females aged 40–49 in 2023. These findings are consistent with concerns that delayed diagnoses may translate into later-stage disease and excess mortality in subsequent years [34]. Similar concerns have been reported from the United States and the United Kingdom, where pandemic-related diagnostic delays were projected to affect future cancer outcomes [26]. Overall, the Hungarian patterns suggest that the impact of the pandemic on cancer care was heterogeneous and depended on age, cancer type, and healthcare system resilience. Because cancer mortality often lags diagnosis by several years, particularly in tumors with improved survival, the absence of a detectable mortality deviation by 2023 should be interpreted cautiously. Therefore, any impact of COVID on the mortality data of later years is yet to be seen [35].

### 4.2. Strengths and Limitations

The strengths of our study include a large nationwide cancer patient cohort from the Hungarian National Health Insurance Fund (NHIF) ensuring robust statistical power and reliability, thorough data cleaning and validation, an extended 9-year pre-pandemic follow-up for detailed trend analysis, and comprehensive outcome assessment from integrating NHIF and Central Statistical Office (CSO) mortality data. Methodological steps to exclude misclassified cases, by cross-referencing cancer-related ICD codes with cancer-specific interventions and mortality, further strengthen validity. However, incidence rates are generally lower than those reported by the Hungarian National Cancer Registry, primarily due to differences in reporting, data processing and case definitions including stricter inclusion criteria, lack of post-mortem diagnoses in the NHIF, and limited clinical detail on tumor subtype and staging. In addition, major healthcare system reforms implemented in the post-pandemic period may have altered diagnostic pathways and healthcare utilization patterns. Since these structural changes could not be disaggregated from COVID-19-related effects within the available aggregated administrative data, residual confounding from non-pandemic system-level factors cannot be excluded [36,37]. For analytical purposes, we defined 2011–2019 as the pre-pandemic period, 2020–2021 as the COVID period and 2022–2023 as the post-COVID period, acknowledging that healthcare recovery was gradual and that this calendar-based definition may not fully capture the pace of recovery across different cancer diagnostic pathways. Accordingly, the post-COVID period should be interpreted as an operational analytical category rather than evidence of complete normalization of healthcare utilization or diagnostic activity.

Another main limitation of the present analysis is the short post-pandemic follow-up, limited to 2022–2023. This timeframe may be insufficient to capture delayed effects on cancer mortality and long-term recovery in incidence, particularly for tumors with long survival times or strong stage-dependent prognosis. Longer follow-up will be needed to determine whether the observed incidence deficits were fully compensated and whether any mortality impact becomes detectable in subsequent years. In addition, because this is an ecological analysis based on administrative incidence data, the observed changes may also reflect shifts in healthcare-seeking behavior, diagnostic intensity, surveillance practices, or coding variability rather than true epidemiological changes alone. Although the long pre-pandemic period allowed us to estimate secular trends, residual confounding by contemporaneous healthcare system changes unrelated to COVID-19 cannot be excluded. Therefore, the present analysis cannot disentangle changes in case detection and healthcare utilization from underlying incidence dynamics. Despite these limitations, the robust dataset and methodology provide important insights into Hungarian cancer epidemiology before and during the COVID-19 pandemic.

## 5. Conclusions

Recent analyses from the Hungarian HUN-CANCER EPI study series show a prolonged and more pronounced decline in cancer incidence during the COVID-19 pandemic compared to most international trends. While several Western countries experienced a rebound in new diagnoses by 2021, Hungary’s cancer incidence remained significantly below pre-pandemic levels through 2022, with only a partial recovery seen in 2023. This delayed rebound was primarily observed in screening-dependent, high-survival cancers. In contrast, for rapidly progressing or symptomatic cancers, such as lung or pancreatic cancer, incidence deficits were not recovered by 2023.

These findings emphasize the need for standardized, long-term surveillance strategies to fully understand and mitigate the pandemic’s lasting impact on cancer care and outcomes and support a conceptual framework for interpreting the effect of the pandemic on cancer detection. Our results regarding the timing and magnitude of cancer incidence declines and rebounds in Hungary provide valuable insights into the resilience and limitations of oncology care systems during crises. Understanding tumor-specific and demographic nuances will inform future health policies, including screening strategy adaptation, diagnostic pathway restoration, and targeted resource allocation.

## Data Availability

Data sharing is available in Supplementary tables and figures, and the authors are happy to share all available data from the study.

## Supplementary Materials

The following supporting information can be downloaded at: https://www.mdpi.com/article/10.3390/cancers18132027/s1, Figure S1: Trends in age-standardized lung cancer incidence (C34) in Hungary by sex, 2011–2023, including average annual change and deviations during COVID and post-COVID periods (ESP 2013). ESP, European Standard Population.; Figure S2: Age-standardized incidence and mortality rates of colorectal cancer (C18–21) by sex in Hungary between 2011 and 2023 (ESP 2013), with percentage changes shown relative to the expected trend based on the pre-COVID period (2011–2019) and specifically change in 2019 versus 2011–2018 period at incidence (green). The figure also highlights changes in incidence during the COVID period (2020–2021) and in the post-COVID years (2022–2023), alongside diverging mortality trends (*relates to significant changes). ESP, European Standard Population.; Figure S3: Age-standardized incidence and mortality rates of breast cancer (C50) in Hungary between 2011 and 2023 (ESP 2013), with percentage changes shown relative to the expected trend based on the pre-COVID period (2011–2019). The figure highlights changes in incidence during the COVID period (2020–2021) and in the post-COVID years (2022–2023), alongside diverging mortality trends (*relates to significant changes). ESP, European Standard Population.; Figure S4: Age-specific cancer incidence rates among females with breast cancer (C50) by different age groups during the pre-COVID (2011–2019), in the COVID (2020–2021) and post-COVID (2022–2023) periods.; Figure S5: Age-standardized incidence and mortality rates of melanoma (C43) in Hungary between 2011 and 2023 (ESP 2013), with percentage changes shown relative to the expected trend based on the pre-COVID period (2011–2019). The figure highlights significant declines in incidence during the COVID period (2020–2021) and a rebound in the post-COVID years (2022–2023), alongside diverging mortality trends by sex. ESP, European Standard Population.; Figure S6: Age-standardized incidence and mortality rates of prostate cancer (C61) in Hungary between 2011 and 2023 (ESP 2013), with percentage changes shown relative to the expected trend based on the pre-COVID period (2011–2019). The figure highlights significant declines in incidence during the COVID period (2020–2021) and a rebound in the post-COVID years (2022–2023), alongside diverging mortality trends. ESP, European Standard Population.; Table S1: Comparison of model estimates: 10-year versus single-year age groupings. Outputs from a Poisson regression model using 10-year age bins were compared against those from an equivalent model fitted on a more granular dataset with single-year age groups. Color-coded columns indicate the relative difference between the two sets of estimates, highlighting strata where the models yielded discordant results.; Table S2: Incidence counts (A) and mortality counts (B) by age, sex, and year of diagnosis; Table S3: Age-specific incidence (A) and mortality (B) rates by sex and year of diagnosis; Table S4: Age-standardized incidence (A) and mortality (B) rates per 100,000 persons based on ESP 1976; Table S5: Age standardized incidence (A) and mortality (B) rates per 100,000 persons based on ESP 2013.

## Abbreviations

The following abbreviations are used in this manuscript:

AAPC	Average Annual Percent Change
CI	Confidence Interval
COVID-19	Coronavirus Disease 2019
CSO	Central Statistical Office
EKU	Egészségügyi Tudományos Tanács (Hungarian National Ethical Committee, referenced as IV/298-2/2022/EKU)
ESP	European Standard Population
HCSO	Hungarian Central Statistical Office
HUN-CANCER EPI	Hungarian Cancer Epidemiology
ICD-10	International Statistical Classification of Diseases and Related Health Problems, 10th Revision
NHIF	National Health Insurance Fund
NHS	National Health Service
NM	Nodular Melanoma
PYs	Linear dichroism
SARS-CoV-2	Severe Acute Respiratory Syndrome Coronavirus 2
SEER	Surveillance, Epidemiology, and End Results
SSM	Superficial Spreading Melanoma
