# Supplementary material for: Cancer Diagnoses and Deaths in Hungary, 2011–2023: Nationwide Trends Before, During, and After the COVID-19 Pandemic
Source: Cancers (Basel). 2026 Jun 23;18(13):2027. doi: 10.3390/cancers18132027 (PMC13359705; doi:10.3390/cancers18132027)
Supplement: Supplementary file 1 [file cancers-18-02027-s001.zip › cancers-4310100-supplementary figures.pptx]

## Slide 1
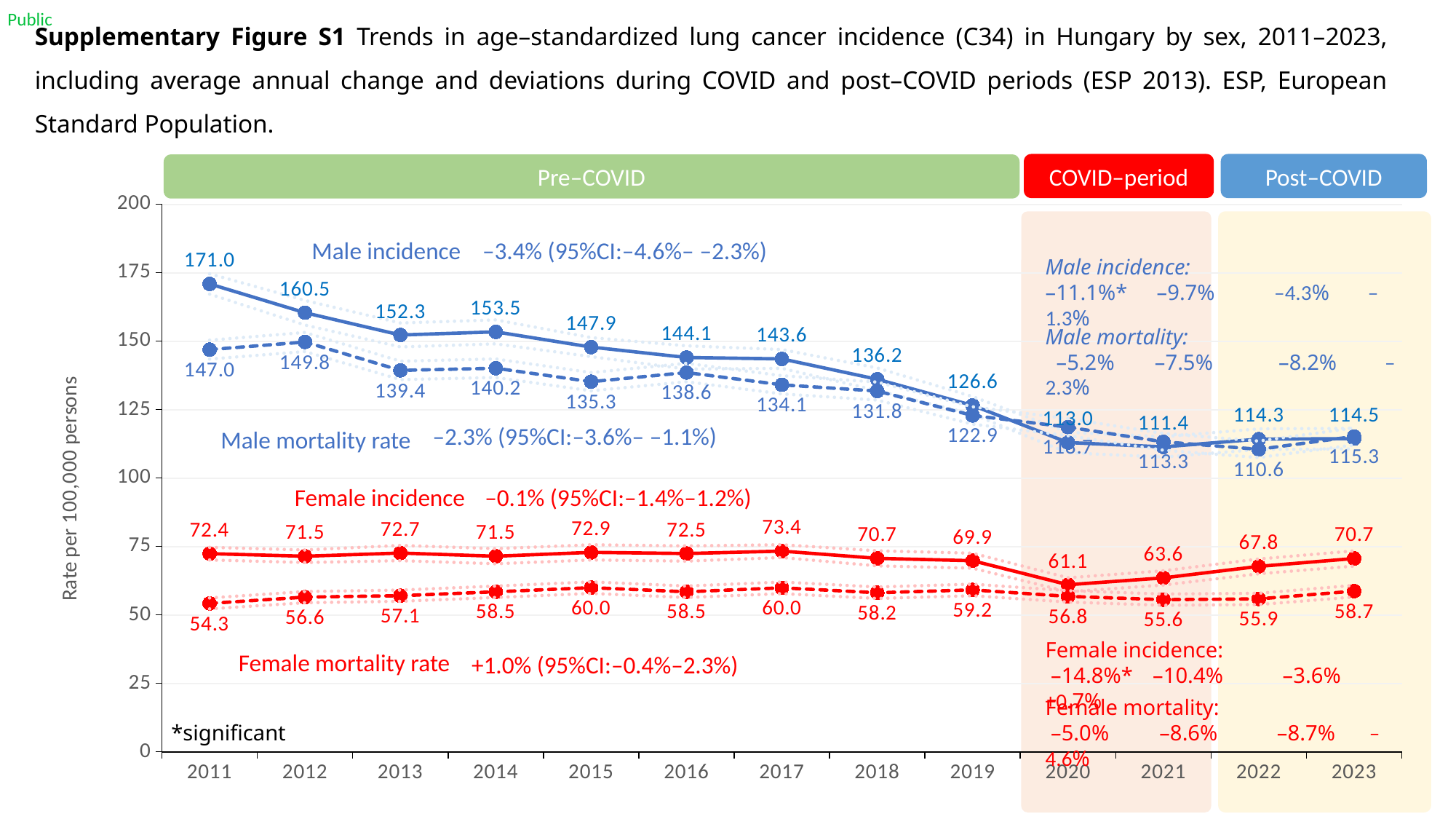

Supplementary Figure S1 Trends in age–standardized lung cancer incidence (C34) in Hungary by sex, 2011–2023, including average annual change and deviations during COVID and post–COVID periods (ESP 2013). ESP, European Standard Population.
### Chart
| Category | | | | | | | | | | | | |
|---|---|---|---|---|---|---|---|---|---|---|---|---|
| 2011 | 170.98107273758 | 72.4353978954282 | 167.260913099042 | 174.701232376118 | 70.1304937105491 | 74.7403020803073 | 146.983879998108 | 150.433526581168 | 143.534233415048 | 54.2645220271437 | 56.2596691441518 | 52.2693749101356 |
| 2012 | 160.460442605216 | 71.4956955051184 | 155.969943010866 | 164.950942199565 | 69.1983363844703 | 73.7930546257665 | 149.773507226633 | 153.262136979892 | 146.284877473375 | 56.5573696957573 | 58.6008290419992 | 54.5139103495154 |
| 2013 | 152.332667451802 | 72.6553587022147 | 147.960111520328 | 156.705223383277 | 69.8774378872538 | 75.4332795171756 | 139.402231461331 | 142.77188076092 | 136.032582161743 | 57.0865916064421 | 59.1428769993309 | 55.0303062135534 |
| 2014 | 153.474856706325 | 71.5029002045828 | 149.086962652307 | 157.862750760342 | 68.7458366538127 | 74.259963755353 | 140.19216936697 | 143.574963569396 | 136.809375164544 | 58.515089695641 | 60.6002555943908 | 56.4299237968911 |
| 2015 | 147.913266554988 | 72.9079210978317 | 144.435949181347 | 151.39058392863 | 70.1226620301224 | 75.693180165541 | 135.273140090934 | 138.59877092849 | 131.947509253379 | 60.0206459472941 | 62.1357287654035 | 57.9055631291846 |
| 2016 | 144.119415937844 | 72.4881874980901 | 139.871849376734 | 148.366982498953 | 69.7091183719305 | 75.2672566242497 | 138.620158354033 | 141.990304768829 | 135.250011939236 | 58.5146234725965 | 60.606831584851 | 56.422415360342 |
| 2017 | 143.59047642008 | 73.3637126412302 | 140.157425502755 | 147.023527337406 | 71.0171780730384 | 75.710247209422 | 134.131759023017 | 137.449968538564 | 130.81354950747 | 59.9531407988396 | 62.0745351878677 | 57.8317464098115 |
| 2018 | 136.15843757935 | 70.7192276918934 | 132.036808947814 | 140.280066210885 | 67.9705162615347 | 73.4679391222521 | 131.844591326427 | 135.134333134177 | 128.554849518678 | 58.1679570779275 | 60.2601403324433 | 56.0757738234118 |
| 2019 | 126.609818942767 | 69.8661935791989 | 123.387545501641 | 129.832092383892 | 67.1340988170655 | 72.5982883413323 | 122.927467813424 | 126.102595330786 | 119.752340296062 | 59.1930142721417 | 61.30540824991 | 57.0806202943734 |
| 2020 | 113.041235099837 | 61.0771157199112 | 109.297424832505 | 116.78504536717 | 58.521546489972 | 63.6326849498504 | 118.677330349568 | 121.799159483267 | 115.555501215869 | 56.8262788931299 | 58.899086698223 | 54.7534710880367 |
| 2021 | 111.446043729217 | 63.576958545598 | 107.727652070947 | 115.164435387487 | 60.9666653881354 | 66.1872517030607 | 113.298775650262 | 116.355003568762 | 110.242547731762 | 55.6400027555183 | 57.6955919396804 | 53.5844135713562 |
| 2022 | 114.283542798956 | 67.7866219220909 | 110.519742741193 | 118.04734285672 | 65.0898960460061 | 70.4833477981757 | 110.564404785646 | 113.587163283977 | 107.541646287316 | 55.9138888764542 | 57.9776102407054 | 53.850167512203 |
| 2023 | 114.465865341061 | 70.6701362800253 | 110.703945969657 | 118.227784712464 | 67.8984820135406 | 73.4417905465099 | 115.27095237045 | 118.363623120583 | 112.178281620316 | 58.7408559623259 | 60.8714743777874 | 56.6102375468643 |COVID–period
Post–COVID
Pre–COVID
Male incidence
–3.4% (95%CI:–4.6%– –2.3%)
Male incidence:
–11.1%*   –9.7% –4.3%   –1.3%
Male mortality:
 –5.2%   –7.5% –8.2%   –2.3%
–2.3% (95%CI:–3.6%– –1.1%)
Male mortality rate
Female incidence
–0.1% (95%CI:–1.4%–1.2%)
Female incidence:
 –14.8%*   –10.4%   –3.6%   +0.7%
Female mortality rate
+1.0% (95%CI:–0.4%–2.3%)
Female mortality:
 –5.0%   –8.6%   –8.7%   –4.6%
*significant

## Slide 2
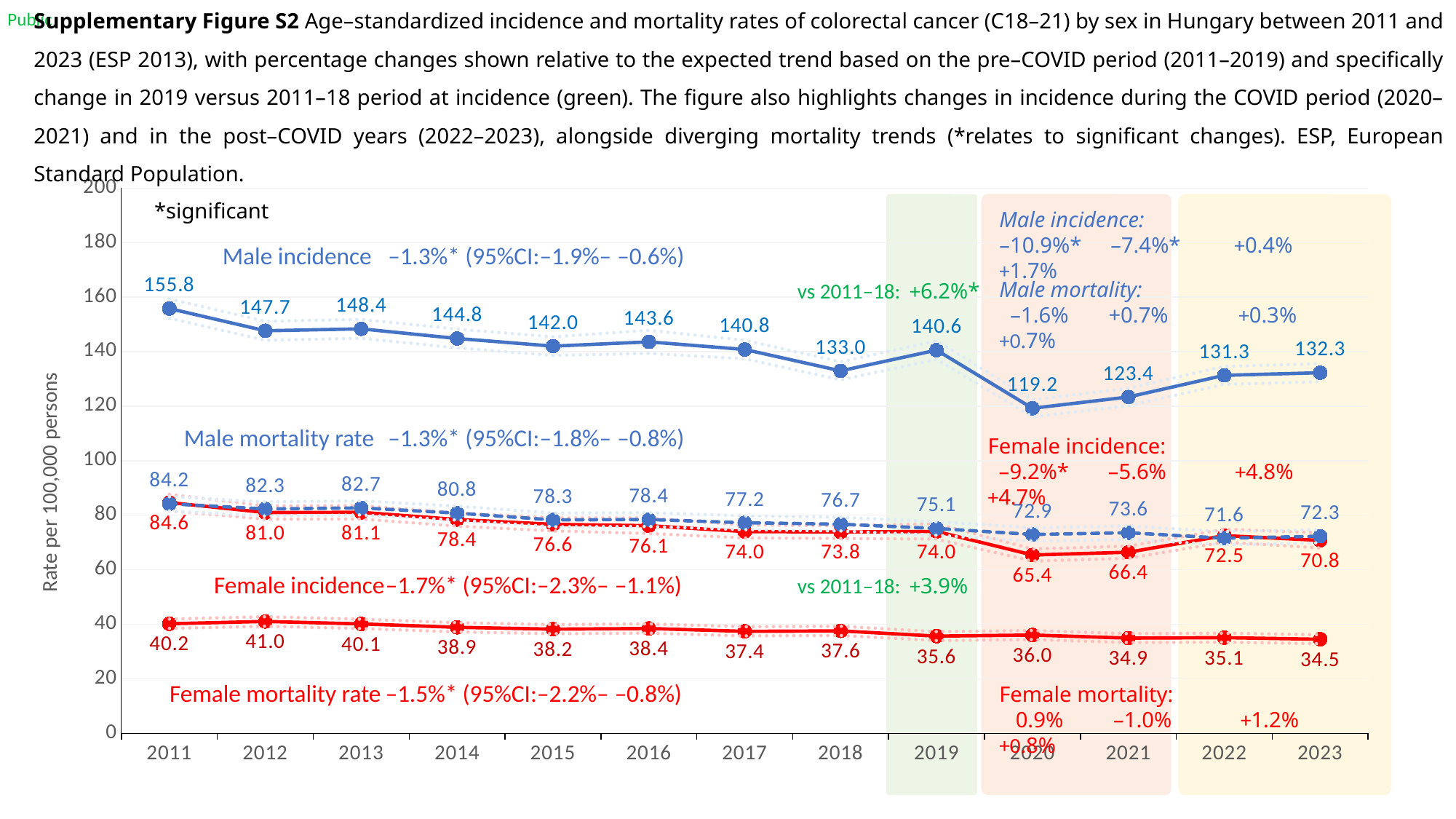

Supplementary Figure S2 Age–standardized incidence and mortality rates of colorectal cancer (C18–21) by sex in Hungary between 2011 and 2023 (ESP 2013), with percentage changes shown relative to the expected trend based on the pre–COVID period (2011–2019) and specifically change in 2019 versus 2011–18 period at incidence (green). The figure also highlights changes in incidence during the COVID period (2020–2021) and in the post–COVID years (2022–2023), alongside diverging mortality trends (*relates to significant changes). ESP, European Standard Population.
### Chart
| Category | | | | | | | | | | | | |
|---|---|---|---|---|---|---|---|---|---|---|---|---|
| 2011 | 155.820809718888 | 84.6259557844146 | 152.26913437611 | 159.372485061665 | 81.6217923784991 | 87.6301191903301 | 84.193833779509 | 86.8054923365552 | 81.5821752224628 | 40.1839156574013 | 41.9009289243064 | 38.4669023904962 |
| 2012 | 147.664148020366 | 80.9899188698877 | 144.200135127423 | 151.128160913308 | 78.5448911281626 | 83.4349466116128 | 82.3036348346565 | 84.8906177914837 | 79.7166518778292 | 41.0213432009308 | 42.7617873493111 | 39.2808990525505 |
| 2013 | 148.377262267502 | 81.0508275824505 | 144.900988266897 | 151.853536268107 | 78.6009578808069 | 83.5006972840942 | 82.6814452960012 | 85.2772808410678 | 80.0856097509346 | 40.1283346433556 | 41.8524993227949 | 38.4041699639163 |
| 2014 | 144.840556840559 | 78.4171445207093 | 141.402217913192 | 148.278895767925 | 76.0035242655699 | 80.8307647758487 | 80.7681765341913 | 83.3365786491645 | 78.1997744192181 | 38.8884362472587 | 40.5884778747647 | 37.1883946197527 |
| 2015 | 142.036826104882 | 76.6453945908455 | 138.629183614148 | 145.444468595616 | 74.25547135265 | 79.035317829041 | 78.2701293959331 | 80.8005352961342 | 75.7397234957319 | 38.2109403403177 | 39.8987298759932 | 36.5231508046421 |
| 2016 | 143.592171645837 | 76.1458628533588 | 139.352370627316 | 147.831972664359 | 73.2975942882239 | 78.9941314184937 | 78.3969234432546 | 80.9321454661232 | 75.861701420386 | 38.4411364989644 | 40.1370917695852 | 36.7451812283436 |
| 2017 | 140.801091580739 | 74.0103291365032 | 137.401501894003 | 144.200681267474 | 71.653483882303 | 76.3671743907033 | 77.2366636890842 | 79.7553461858338 | 74.7179811923346 | 37.4061531464461 | 39.0820062724131 | 35.7303000204791 |
| 2018 | 132.965683673441 | 73.8395521756471 | 129.662003456404 | 136.269363890478 | 71.4825049163549 | 76.1965994349393 | 76.7134271427198 | 79.2234991952255 | 74.203355090214 | 37.5642056222941 | 39.2456777881839 | 35.8827334564043 |
| 2019 | 140.575689631036 | 74.0419486675315 | 137.180582817562 | 143.97079644451 | 71.2294516972502 | 76.8544456378127 | 75.1339828074458 | 77.6168803273087 | 72.6510852875829 | 35.6419060424661 | 37.281254657157 | 34.0025574277753 |
| 2020 | 119.24033967294 | 65.4069452957055 | 116.111123088084 | 122.369556257796 | 63.183237516476 | 67.630653074935 | 72.9342148436151 | 75.3820959837272 | 70.486333703503 | 36.0417524414239 | 37.6926971541432 | 34.3908077287045 |
| 2021 | 123.378221627208 | 66.4155388840639 | 120.189104498486 | 126.567338755931 | 64.1698262110378 | 68.66125155709 | 73.5840470299084 | 76.0475397617584 | 71.1205542980584 | 34.9245711435316 | 36.5533168984747 | 33.2958253885885 |
| 2022 | 131.327318532111 | 72.5061605610214 | 128.033283736296 | 134.621353327926 | 70.1562971879617 | 74.8560239340812 | 71.5600705339537 | 73.9923638071692 | 69.1277772607381 | 35.0835937927765 | 36.7184836542731 | 33.44870393128 |
| 2023 | 132.279767866103 | 70.7735662227267 | 128.967057411199 | 135.592478321008 | 67.9998858928991 | 73.5472465525543 | 72.2986955705843 | 74.7485054277734 | 69.8488857133953 | 34.4986889221894 | 36.131701395962 | 32.8656764484168 |*significant
Male incidence:
–10.9%*   –7.4%* +0.4%   +1.7%
Male incidence
–1.3%* (95%CI:–1.9%– –0.6%)
Male mortality:
 –1.6%   +0.7% +0.3%   +0.7%
 vs 2011–18: +6.2%*
Male mortality rate
–1.3%* (95%CI:–1.8%– –0.8%)
Female incidence:
 –9.2%*   –5.6%   +4.8%   +4.7%
Rate per 100,000 persons
Female incidence
–1.7%* (95%CI:–2.3%– –1.1%)
 vs 2011–18: +3.9%
–1.5%* (95%CI:–2.2%– –0.8%)
Female mortality rate
Female mortality:
 0.9%   –1.0%   +1.2%   +0.8%

## Slide 3
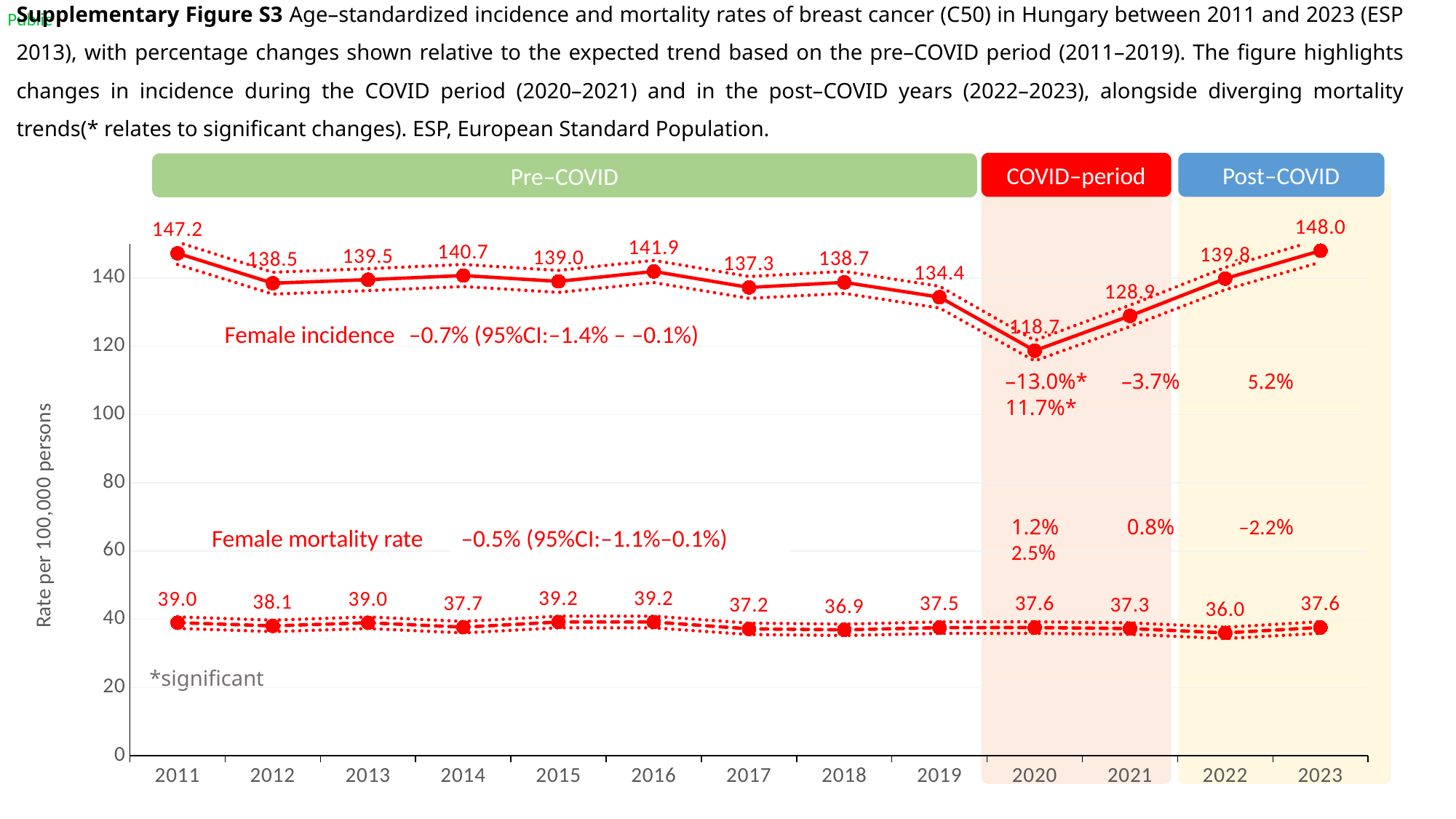

Supplementary Figure S3 Age–standardized incidence and mortality rates of breast cancer (C50) in Hungary between 2011 and 2023 (ESP 2013), with percentage changes shown relative to the expected trend based on the pre–COVID period (2011–2019). The figure highlights changes in incidence during the COVID period (2020–2021) and in the post–COVID years (2022–2023), alongside diverging mortality trends(* relates to significant changes). ESP, European Standard Population.
### Chart
| Category | | | | | | |
|---|---|---|---|---|---|---|
| 2011 | 147.242073220746 | 143.957107862494 | 150.527038578997 | 39.0095761670731 | 40.7013242421536 | 37.3178280919925 |
| 2012 | 138.493758508488 | 135.297378507814 | 141.690138509161 | 38.0593394534266 | 39.7357956502188 | 36.3828832566344 |
| 2013 | 139.520216557471 | 136.306883618822 | 142.733549496121 | 38.9830379565227 | 40.6824297009205 | 37.283646212125 |
| 2014 | 140.737617512502 | 137.505158315035 | 143.970076709969 | 37.6876927230697 | 39.3612928467346 | 36.0140925994049 |
| 2015 | 139.014909060185 | 135.797279597374 | 142.232538522995 | 39.2001378769241 | 40.909625952001 | 37.4906498018472 |
| 2016 | 141.93013792673 | 138.673056091648 | 145.187219761812 | 39.1979481630885 | 40.9105101800216 | 37.4853861461554 |
| 2017 | 137.250206859139 | 134.04169288061 | 140.458720837668 | 37.1983152760817 | 38.8695079263164 | 35.527122625847 |
| 2018 | 138.730062431683 | 135.50031920414 | 141.959805659226 | 36.9086127456231 | 38.575352765054 | 35.2418727261922 |
| 2019 | 134.405272391431 | 131.223388223103 | 137.587156559759 | 37.54709032079 | 39.2296669899677 | 35.8645136516122 |
| 2020 | 118.722134182975 | 115.727003678136 | 121.717264687815 | 37.5732007797182 | 39.2588428038636 | 35.8875587555727 |
| 2021 | 128.918931439467 | 125.791110873279 | 132.046752005654 | 37.2779999816073 | 38.9607087023624 | 35.5952912608522 |
| 2022 | 139.834314569704 | 136.572076284899 | 143.096552854508 | 36.0075429491469 | 37.6638131959873 | 34.3512727023065 |
| 2023 | 148.014996384182 | 144.634392052939 | 151.395600715426 | 37.5962380417828 | 39.3009604640906 | 35.8915156194751 |COVID–period
Post–COVID
Pre–COVID
Female incidence
–0.7% (95%CI:–1.4% – –0.1%)
–13.0%*   –3.7% 5.2%   11.7%*
1.2%   0.8%   –2.2%   2.5%
Female mortality rate
–0.5% (95%CI:–1.1%–0.1%)
*significant

## Slide 4
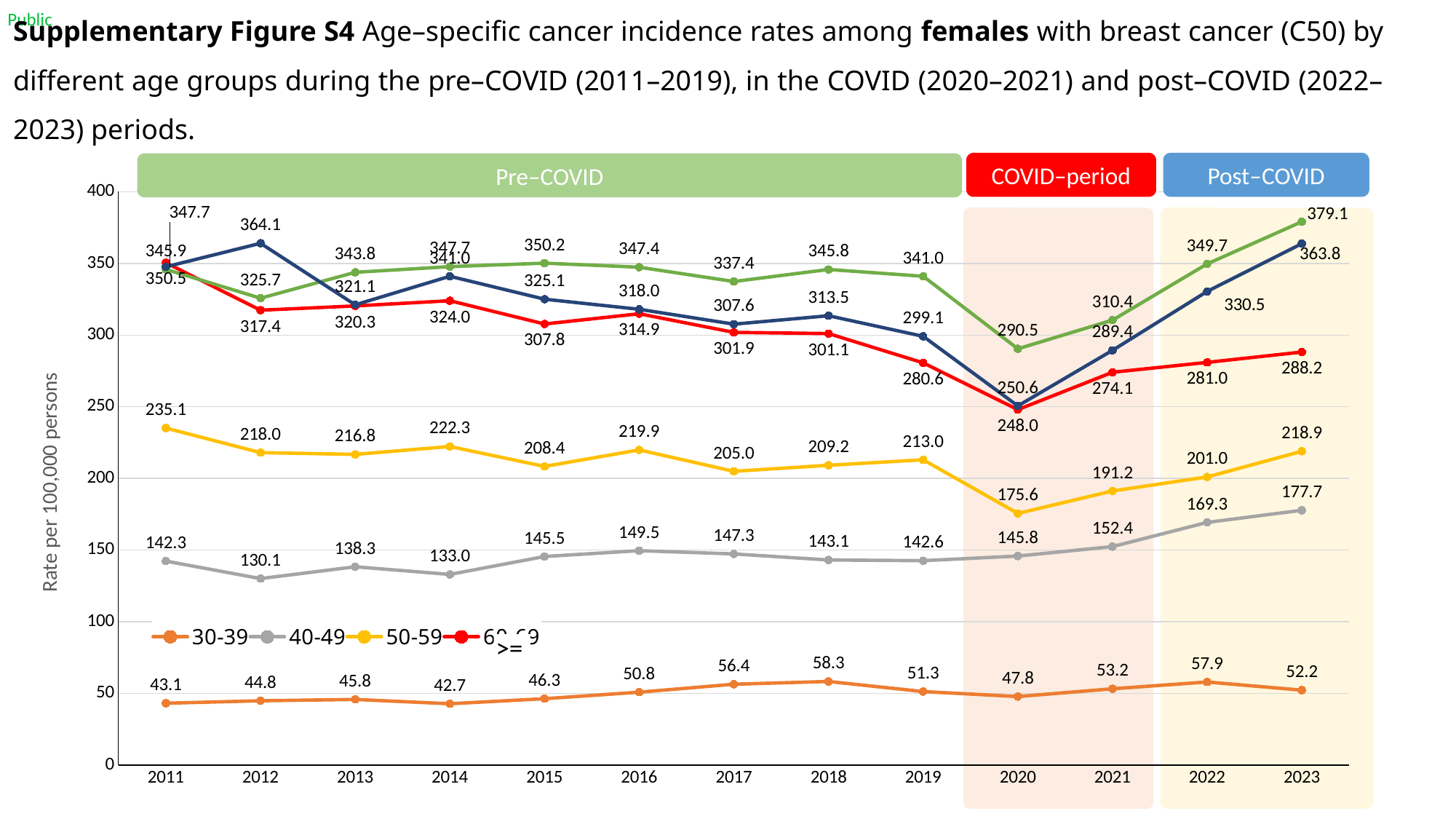

Supplementary Figure S4 Age–specific cancer incidence rates among females with breast cancer (C50) by different age groups during the pre–COVID (2011–2019), in the COVID (2020–2021) and post–COVID (2022–2023) periods.
COVID–period
Post–COVID
Pre–COVID
### Chart
| Category | 30-39 | 40-49 | 50-59 | 60-69 | 70-79 | 80<= |
|---|---|---|---|---|---|---|
| 2011 | 43.1335990497959 | 142.294397273357 | 235.120310139346 | 350.456715501095 | 345.876144497039 | 347.652647233686 |
| 2012 | 44.8411136316341 | 130.073600978249 | 217.993037679155 | 317.379970515055 | 325.745822205425 | 364.061203164125 |
| 2013 | 45.7591707915291 | 138.333856990753 | 216.767568130172 | 320.335156192913 | 343.801133178464 | 321.129153914845 |
| 2014 | 42.7468766550972 | 132.988728202137 | 222.290357197608 | 323.984724379203 | 347.749424499192 | 341.029272818293 |
| 2015 | 46.2783017680828 | 145.496911986792 | 208.35554104022 | 307.750120313114 | 350.20150723982 | 325.065748860494 |
| 2016 | 50.8178173965843 | 149.539897778798 | 219.901734353372 | 314.874877055178 | 347.367657956632 | 318.006045130721 |
| 2017 | 56.3721711900422 | 147.306177456931 | 204.953058880474 | 301.895351945713 | 337.393891415798 | 307.641251140795 |
| 2018 | 58.2990852187658 | 143.066310590516 | 209.16457209147 | 301.055802018093 | 345.770486019229 | 313.512624393453 |
| 2019 | 51.253229272779 | 142.578835546502 | 212.98967190544 | 280.628326961562 | 341.037762146346 | 299.139526291891 |
| 2020 | 47.7693035105564 | 145.808763451114 | 175.567487292595 | 247.987547502406 | 290.4522783885 | 250.615667344746 |
| 2021 | 53.2022762035578 | 152.406085503394 | 191.236300636546 | 274.052699033096 | 310.440805471054 | 289.355838787461 |
| 2022 | 57.9332796243554 | 169.278738796217 | 201.023195809157 | 280.951804888063 | 349.695694843138 | 330.469895811508 |
| 2023 | 52.2404563112062 | 177.743705129232 | 218.933177687893 | 288.173215467892 | 379.103300170925 | 363.80897265418 |
Rate per 100,000 persons
>=

## Slide 5
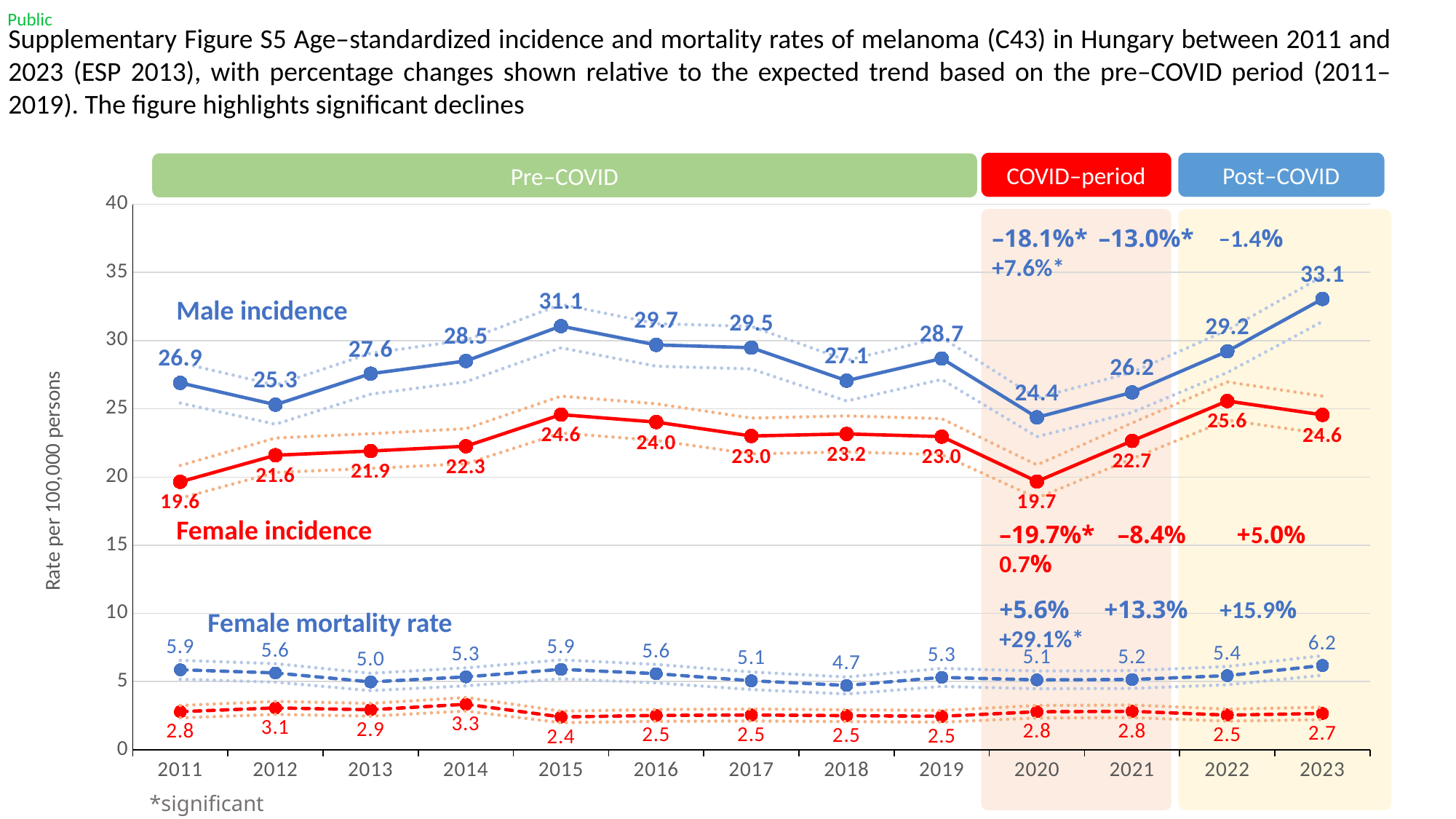

Supplementary Figure S5 Age–standardized incidence and mortality rates of melanoma (C43) in Hungary between 2011 and 2023 (ESP 2013), with percentage changes shown relative to the expected trend based on the pre–COVID period (2011–2019). The figure highlights significant declines
COVID–period
Post–COVID
Pre–COVID
### Chart
| Category | | | | | | | | | | | | |
|---|---|---|---|---|---|---|---|---|---|---|---|---|
| 2011 | 26.9021067124821 | 19.6463676068657 | 25.4254012655715 | 28.3788121593927 | 18.4456710449048 | 20.8470641688266 | 5.86517781795524 | 6.55476155644625 | 5.17559407946422 | 2.79466939278471 | 3.24756066409903 | 2.34177812147038 |
| 2012 | 25.3039472185904 | 21.5976609118637 | 23.869111973395 | 26.7387824637858 | 20.3346686109726 | 22.8606532127548 | 5.63434521574586 | 6.3114756664203 | 4.95721476507142 | 3.06916837273051 | 3.54532292432171 | 2.59301382113931 |
| 2013 | 27.5759228480967 | 21.9047330769401 | 26.0763833868147 | 29.0754623093786 | 20.6307556212013 | 23.1787105326789 | 4.97195876708323 | 5.60876267858073 | 4.33515485558574 | 2.9327052404322 | 3.39890084019816 | 2.46650964066624 |
| 2014 | 28.5069548007022 | 22.2574781391117 | 26.9806831443855 | 30.0332264570188 | 20.971233972414 | 23.5437223058094 | 5.34556416437162 | 6.00656727298436 | 4.68456105575888 | 3.33625679365997 | 3.8342876696651 | 2.83822591765483 |
| 2015 | 31.0649223255747 | 24.5767665749651 | 29.470402723529 | 32.6594419276203 | 23.2230853200852 | 25.9304478298451 | 5.89609427686858 | 6.59084914673029 | 5.20133940700687 | 2.40713292297471 | 2.83082683758931 | 1.98343900836011 |
| 2016 | 29.6860735912137 | 24.0278439464086 | 28.1256277495981 | 31.2465194328293 | 22.6869173845238 | 25.3687705082933 | 5.58073089754474 | 6.25739044561272 | 4.90407134947676 | 2.51808700797963 | 2.95222687859655 | 2.0839471373627 |
| 2017 | 29.4826126364665 | 23.0098676712176 | 27.9261152119556 | 31.0391100609773 | 21.695391368374 | 24.3243439740613 | 5.05944630313871 | 5.70431301937869 | 4.41457958689874 | 2.54764345216455 | 2.98507435716143 | 2.11021254716768 |
| 2018 | 27.0640390978596 | 23.1594228040754 | 25.5727746303183 | 28.5553035654008 | 21.8390464661817 | 24.4797991419691 | 4.71343791821202 | 5.33584682804355 | 4.09102900838049 | 2.49996350263844 | 2.9338194633397 | 2.06610754193717 |
| 2019 | 28.6897486652786 | 22.9633264123309 | 27.1551144890563 | 30.2243828415009 | 21.6473872965571 | 24.2792655281048 | 5.30580767980751 | 5.96584430048445 | 4.64577105913057 | 2.45490243531516 | 2.88521106350352 | 2.0245938071268 |
| 2020 | 24.3746313266204 | 19.6702863390941 | 22.9591661307508 | 25.7900965224901 | 18.4505373098353 | 20.8900353683529 | 5.12499306715504 | 5.77410353787993 | 4.47588259643015 | 2.78722087610293 | 3.24640567074716 | 2.3280360814587 |
| 2021 | 26.2060103913389 | 22.6506880816866 | 24.7355178151648 | 27.676502967513 | 21.338925870619 | 23.9624502927541 | 5.15117213585974 | 5.80319247067695 | 4.49915180104253 | 2.81705291063127 | 3.27970549412993 | 2.3544003271326 |
| 2022 | 29.2147351638408 | 25.5736870583434 | 27.6602967016335 | 30.7691736260482 | 24.1777887091886 | 26.9695854074981 | 5.43591040007768 | 6.10650545363109 | 4.76531534652427 | 2.54115990278839 | 2.98123129669295 | 2.10108850888382 |
| 2023 | 33.0557937748444 | 24.5573396245152 | 31.398970442563 | 34.7126171071257 | 23.1794943255502 | 25.9351849234801 | 6.180295705503 | 6.8967944027197 | 5.46379700828629 | 2.66079065763807 | 3.11437986063759 | 2.20720145463855 |
–18.1%* –13.0%* –1.4%   +7.6%*
Male incidence
Rate per 100,000 persons
Female incidence
–19.7%*   –8.4% +5.0%   0.7%
+5.6% +13.3% +15.9%   +29.1%*
Female mortality rate
*significant

## Slide 6
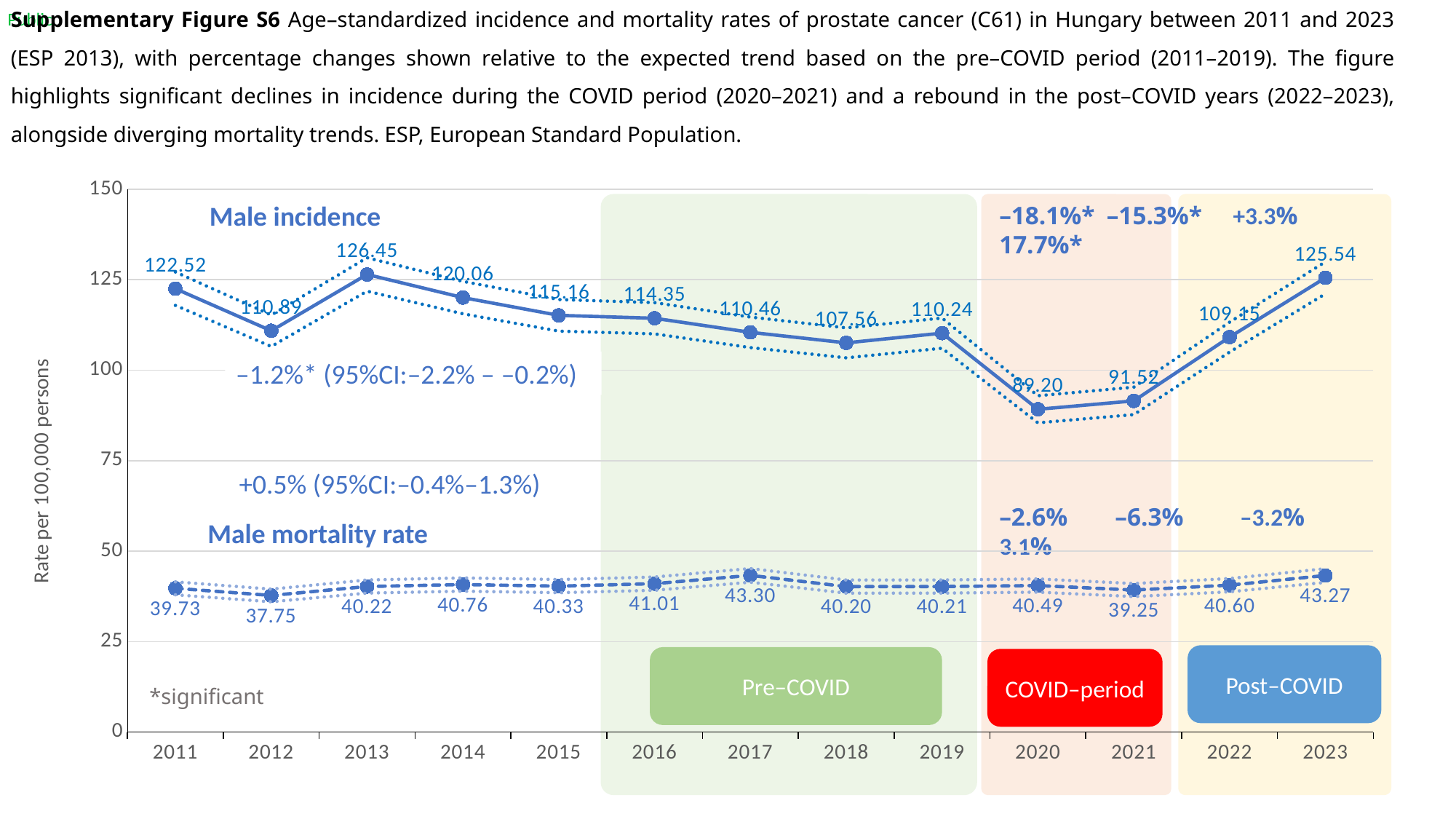

Supplementary Figure S6 Age–standardized incidence and mortality rates of prostate cancer (C61) in Hungary between 2011 and 2023 (ESP 2013), with percentage changes shown relative to the expected trend based on the pre–COVID period (2011–2019). The figure highlights significant declines in incidence during the COVID period (2020–2021) and a rebound in the post–COVID years (2022–2023), alongside diverging mortality trends. ESP, European Standard Population.
### Chart
| Category | | | | | | | |
|---|---|---|---|---|---|---|---|
| 2011 | 122.524119895443 | 117.903480722269 | 127.144759068616 | 39.7273657326041 | 41.5217595183751 | 37.9329719468332 | None |
| 2012 | 110.893916695449 | 106.551061045134 | 115.236772345763 | 37.7533435779002 | 39.5058457615186 | 36.0008413942818 | None |
| 2013 | 126.451124765735 | 121.83006683659 | 131.072182694881 | 40.2238565232508 | 42.0348098220715 | 38.4129032244301 | None |
| 2014 | 120.063382261781 | 115.583264989262 | 124.543499534301 | 40.7622310463121 | 42.5872138542414 | 38.9372482383828 | None |
| 2015 | 115.15773259168 | 110.80069189782 | 119.51477328554 | 40.3310506272061 | 42.1477972686165 | 38.5143039857957 | None |
| 2016 | 114.352951479615 | 110.039487792055 | 118.666415167175 | 41.0140014601198 | 42.8480624965781 | 39.1799404236615 | None |
| 2017 | 110.463734195098 | 106.247070020871 | 114.680398369324 | 43.3012291486088 | 45.1874194947959 | 41.4150388024218 | None |
| 2018 | 107.557752904174 | 103.417482852374 | 111.698022955975 | 40.2019477945552 | 42.0193597195459 | 38.3845358695645 | None |
| 2019 | 110.240980208416 | 106.069636550455 | 114.412323866377 | 40.2118668775579 | 42.0286102449981 | 38.3951235101177 | None |
| 2020 | 89.2023094090416 | 85.4613964923566 | 92.9432223257266 | 40.4913661926373 | 42.3155826696879 | 38.6671497155866 | None |
| 2021 | 91.517948823073 | 87.73084608805 | 95.305051558096 | 39.2468515263238 | 41.0462877794686 | 37.4474152731791 | None |
| 2022 | 109.148314768396 | 105.014815476543 | 113.281814060249 | 40.5964466663195 | 42.4287266793423 | 38.7641666532967 | None |
| 2023 | 125.537608513177 | 121.115605667124 | 129.95961135923 | 43.2651843499858 | 45.1605783787565 | 41.3697903212152 | None |Male incidence
–18.1%*  –15.3%* +3.3%   17.7%*
–1.2%* (95%CI:–2.2% – –0.2%)
+0.5% (95%CI:–0.4%–1.3%)
–2.6%   –6.3%   –3.2%   3.1%
Male mortality rate
Post–COVID
Pre–COVID
COVID–period
*significant
